# Cryptochrome Interacts With Actin and Enhances Eye-Mediated Light Sensitivity of the Circadian Clock in *Drosophila melanogaster*

**DOI:** 10.3389/fnmol.2018.00238

**Published:** 2018-07-18

**Authors:** Matthias Schlichting, Dirk Rieger, Paola Cusumano, Rudi Grebler, Rodolfo Costa, Gabriella M. Mazzotta, Charlotte Helfrich-Förster

**Affiliations:** ^1^Neurobiology and Genetics, Biocenter, Theodor-Boveri-Institute, University of Würzburg, Würzburg, Germany; ^2^Howard Hughes Medical Institute and National Center for Behavioral Genomics, Department of Biology, Brandeis University, Waltham, MA, United States; ^3^Department of Biology, University of Padova, Padova, Italy

**Keywords:** *Drosophila melanogaster*, cryptochrome, F-actin, phototransduction, activity rhythms

## Abstract

Cryptochromes (CRYs) are a class of flavoproteins that sense blue light. In animals, CRYs are expressed in the eyes and in the clock neurons that control sleep/wake cycles and are implied in the generation and/or entrainment of circadian rhythmicity. Moreover, CRYs are sensing magnetic fields in insects as well as in humans. Here, we show that in the fruit fly *Drosophila melanogaster* CRY plays a light-independent role as “assembling” protein in the rhabdomeres of the compound eyes. CRY interacts with actin and appears to increase light sensitivity of the eyes by keeping the “signalplex” of the phototransduction cascade close to the membrane. By this way, CRY also enhances light-responses of the circadian clock.

## Introduction

Nearly all living organisms use daily patterns of day and night to entrain their endogenous circadian clocks. These responses utilize photic input from both visual photoreceptors and non-visual photopigments (reviewed in Golombek and Rosenstein, 2010; Johnsson et al., 2015). Cryptochromes (CRYs; from the Greek κρυπτόχρώμα, *hidden color*) are a class of flavoproteins, non-visual photopigments present in plants and animals, which sense blue light. CRYs are involved in the generation and/or synchronization of circadian rhythms of plants and animals, in developmental processes in plants and in the sensing of magnetic fields in a number of species (Yoshii et al., 2009; Gegear et al., 2010; Chaves et al., 2011; Foley et al., 2011; Fedele et al., 2014). The two principal types of CRYs are the light-sensitive plant/insect type 1 CRY and the mammalian type 2 CRY; the latter is a component of the molecular circadian clockwork and retains light responsiveness only under special conditions (Griffin et al., 1999; Kume et al., 1999; Hoang et al., 2008; Fedele et al., 2014). However, mammals own multiple CRYs of the same type and some arthropods (e.g., mosquitoes, butterflies and krill) have both types of CRYs (Zhu et al., 2005; Yuan et al., 2007; Biscontin et al., 2017).

The fruit fly *Drosophila melanogaster* possesses a single form of type 1 CRY, which appears to have different functions. (1) In *Drosophila* circadian clock neurons, CRY acts as circadian photopigment (Emery et al., 1998, 2000; Stanewsky et al., 1998); upon light-activation, it interacts with the clock protein Timeless (TIM) and provokes its degradation via the proteasomal pathway, therefore resetting the molecular clock (Ceriani et al., 1999; Peschel et al., 2009). (2) In peripheral tissues, including the compound eyes, CRY appears to be an integral component of the molecular clock (Ivanchenko et al., 2001; Krishnan et al., 2001; Collins et al., 2004). (3) In the compound eyes and in a clock neuron subgroup, CRY is additionally associated with the cytoplasmic membrane and appears to interfere with the phototransduction cascade (Mazzotta et al., 2013) and with light-induced membrane depolarization (Fogle et al., 2011, 2015). (4) In the lamina, CRY seems to be involved in the degradation of the presynaptic protein Bruchpilot (BRP), therefore contributing to visual plasticity (Damulewicz et al., 2017).

The function of *Drosophila* CRY in the photoreceptor cells of the compound eyes is so far not well understood. In its C-terminus, CRY carries several protein-protein interaction motifs, including two class III PDZ-binding motifs that play a role in the assembly of large protein complexes involved in signaling processes (PDZ = **P**ostsynaptic density protein 95, ***D**rosophila* disk large tumor suppressor, **Z**onula occludens-1 protein; Hemsley et al., 2007; Mazzotta et al., 2013). In the photoreceptor cells, CRY interacts through its PDZ binding motifs in a light-dependent manner with the scaffolding protein INAD (**I**nactivation **N**o **A**fterpotential **D**) which seems, in turn, to enable interaction between CRY and other phototransduction components (Mazzotta et al., 2013). INAD is important to gather the components of the phototransduction cascade at the membrane of the rhabdomeres and it is bound to F-actin filaments via myosin III (NINAC; Montell, 1999). Especially in the dark, INAD binds via its PDZ-domains 4/5 to TRP-channels and keeps them in the rhabdomeres—ready for activation, whereas after light-adaptation TRP channels move into the cell body (Montell, 2007). Most interestingly, CRY appeared to enhance photosensitivity mainly during the night perhaps by enhancing the interaction between INAD, NINAC and F-actin and hence increasing the activation of TRP channels (Mazzotta et al., 2013). However, this hypothesis limps, because the CRY-INAD interaction has only been found after light exposure and it has not yet been demonstrated that CRY is present in the rhabdomeres. Furthermore, if CRY is indeed involved in photoreception, one should also see differences in fly daily activity patterns when CRY is missing in the compound eyes. The compound eyes have been shown to fine-tune daily activity according to fluctuations in environmental light (Schlichting et al., 2014, 2015). In particular, they seem responsible for setting the ratio of diurnal/nocturnal activity. Flies generally prefer being active at low light intensities and consequently reduce diurnal activity with increasing daylight intensity (Rieger et al., 2007). This response is solely mediated by the compound eyes with a special importance of photoreceptor cells 1-6 (Schlichting et al., 2014, 2015).

Here, we show that CRY is present in the rhabdomeres of all photoreceptor cells, that it interacts with F-actin and may therefore enhance the binding of the phototransduction cascade signaling components to the rhabdomere cytoskeleton. In contrast to the CRY/INAD interaction, the CRY/F-actin binding is light-independent, possibly retaining the signaling components close to the membrane and ready for activation during day and night. Indeed, CRY in the rhabdomeres is not degraded by light, thus permitting the interaction with the signaling components even during long lasting light-exposure. Flies lacking CRY (*cry*^01^ mutants) shift less activity from the day into the night in response to increasing day-light intensities, suggesting that the compound eyes of such flies are less light-sensitive. The wild-type (WT) behavior is fully rescued by expressing CRY in photoreceptor cells R1–6. The role of CRY in enhancing light sensitivity appears to be largely independent of its photoreceptive function, because it persists in red light by which CRY cannot be excited: *cry*^01^ mutants need significantly longer to follow phase-shifts of red light-dark cycles than WT flies and this behavior can be partially rescued by expressing CRY in photoreceptor cells R1–6. We propose a model for CRY action in the eyes that, given the ability of human CRY to interact with actin, might also apply in humans.

## Materials and Methods

### Fly Stocks

To eliminate genetic background effects, *cry*^01^ mutants (Dolezelova et al., 2007) were back-crossed to WT “CantonS” (WT_CantonS_) or to WT “Lindelbach” (WT_Lindelbach_; Schlichting et al., 2014) for five generations and later compared to the relevant WT strains. Rescue experiments were conducted with *ninaE-gal4* (Bloomington #30540) and *uas*-*cry* (Emery et al., 1998) crossed into the *cry*^01^ background. As controls served the offspring of crosses between *ninaE-gal4* and *uas-cry* flies and the *cry*^01^ mutants, respectively. Co-immunoprecipitation (Co-IP) was performed with *yw;tim-gal4*/+*; uas-HAcry*/+ (Dissel et al., 2004).

### Co-immunoprecipitation and 2D SDS PAGE

Co-immunoprecipitation was performed as in Mazzotta et al. (2013). The 2D electrophoresis was performed according to Khoudoli et al. (2004), with some modifications. Protein complexes were solubilized by heat treatment (5 min at 95°C) in presence of 100 mM DTT and 0.2% SDS, precipitated in 80% acetone at −20°C and solubilized for 6 h in resuspension buffer (30 mM Tris Base, 7 M Urea, 2 M Thiourea, 1.2% CHAPS, 0.14% ASB14, 0.25% Ampholytes, 43 mM DTT), with the addition of 60 mM Acrylamide after 3 h, in order to alkylate the proteins (Mineki et al., 2002). Isoelectric focusing (IEF) was performed in 7 cm IPG strips of pH range 4–7 (ReadyStrip™_Bio-rad); strips have been passively rehydrated for 16 h and then iso-electro focused by a two-phase protocol: 30 min at 250 V, 3 h and 30 min at 5500 V and 500 V until the complete focusing. After IEF, strips were equilibrated in Equilibration buffer (50 mM Bis-Tris pH 6.4, 6 M Urea, 30% (w/v) glycerol, 2% SDS) containing 50 mM DTT for 20 min and 360 mM Acrylamide for further 20 min. Strips were then placed on a 4%–12% pre-cast “ZOOM NuPAGE gel” (Invitrogen^®^) with the help of a 0.5% agarose matrix and run at room temperature at 50 V.

### Protein Identification by Mass Spectrometry

After separation on the gel, Coomassie-stained protein spots were excised and in-gel digested, as previously described (Wilm et al., 1996; Mazzotta et al., 2013). MALDI-TOF and LC-MS/MS data were analyzed by the online MASCOT software (Matrix Science^1^) against the *Drosophila* sequences of the Swiss-Prot database (release 2012_04).

### Yeast-Two-Hybrid Assays

The experiments were performed in the EGY48 yeast strain (*MAT*α, *ura*3, *trp*1, *his*3, 3*LexA-operator-LEU*). Full-length hCRY2 and dCRY were fused to the LexA moiety in the bait vector (pEG202), while full-length hActin-Beta, dActin-5C and dActin-57B were fused to the “acid-blob” portion of the prey vector (pJG4–5; Golemis and Brent, 1997).

The full-length h*Cry2* coding sequence was amplified from pSO2002 plasmid (pSO2002 was a gift from Aziz Sancar_Addgene plasmid #25842; Ozgur and Sancar, 2003). The full-length h*Actin-Beta* coding sequence was amplified from cDNA retro-transcribed from the Universal Human Reference RNA, a pool of total RNA from 10 human cell lines (Agilent Technologies, Santa Clara, CA, USA). The full-length d*Actin-5C* and d*Actin-57B* coding sequences were amplified from cDNA extracted from heads of *w*^1118^ flies. The primers used are listed in Supplementary Table S1; all the cloning have been performed by using the In-Fusion^®^ HD Cloning Kit (Clontech). The constructs were fully sequenced to assess the in-frame insertion of the cDNA and to control for unwanted mutations. The reliable expression of bait and prey fusions was confirmed by immunoblot (Supplementary Figure S1). Protein extracts were obtained as in Ausbel (1998), subjected to SDS/PAGE (NuPAGE-Invitrogen), and probed with specific anti-LexA (AbCam; 1:3.000) and anti-HA (Sigma; 1:5.000) antibodies. Expected molecular weights for the tested fusions are listed in Supplementary Table S2.

Quantification of β-galactosidase activity was performed in liquid culture as in Ausbel (1998), either in dark or under a white saturating light (10,000 lx), and the experiment was repeated three times. Statistic analysis was performed with Graphpad Prism v4 using one-way ANOVA followed by Tukey’s multiple comparisons test.

### Immunostaining of Retinas and Brains

Retinas were dissected from male flies at the age of 6–9 days. After raising the flies either in constant darkness, in constant darkness followed by a 2 h exposure to white LED light (1000 lux) or 1 h before lights-off (Zeitgeber Time ZT11) and lights-on (ZT23), respectively, in regular 12:12 h light-dark cycles (500 lux) they were immediately fixed in 4% paraformaldehyde (PFA) in phosphate buffered saline (PBS; pH = 7.4) for 2.75 h in darkness. Afterwards retinas were dissected in PBS with 0.1% Triton X-100 (PBST; pH = 7.4). Blocking, washing and incubation with the primary and secondary antibody was performed analogous to Hsiao et al. (2012) with the modification of a 2-day incubation in the primary antibody solution. The primary antibody solutions contained 5% normal goat serum, PBST and antibodies against CRY (1:2000; Yoshii et al., 2008) and Rh1 (1:30; 4C5, Developmental Studies Hybridoma Bank, Iowa City, IA, USA). For visualization of CRY and Rh1, secondary fluorescent antibodies (Alexa Fluor 555 nm and 647 nm, respectively) were applied at a dilution of 1:200 overnight. For visualizing actin, Phalloidin conjugated with ALEXA Fluor 488 nm (1:200) was added to the solution with the secondary antibodies. For E3 ubiquitin ligase (UBE3A) staining a primary UBE3A antibody was applied at 1:1000 (Lu et al., 2009), visualized with ALEXA Fluor 555 nm (1:200) and co-stained with Phalloidin-conjugated ALEXA Fluor 488 nm (1:200). The latter stainings were done at ZT11 and ZT23 during the regular light-dark cycle.

Brains were dissected in parallel from the same heads of which the retinas were dissected at ZT11 and ZT23 of the light-dark cycle. They were immunostained with anti-CRY (1:2000) and anti-Pigment-Dispersing Factor (PDF, 1:2000; C7, Developmental Studies Hybridoma Bank, Iowa City, IA, USA) following the same procedure and incubation time as for the retinas. Secondary antibodies were Alexa Fluor 555 nm for visualizing CRY and Alexa Fluor 647 nm for visualizing PDF.

### Microscopy and Image Analysis for CRY Staining Level

After mounting on glass slides with Vectashield (Vector Laboratories, Burlingame, CA, USA) image stacks from retinas were recorded using the laserscanning microscope (Leica TSC SPE with Leica DM 5500 Q microscope, Leica, Germany) with a 20× glycerol objective (NA 0.6). Confocal settings for CRY (555 nm) were the following: pinhole 1; 35% laser, 700 gain, −0.1 offset, 2 μm section thickness, 4.5 magnification for the retinas and 1.5 magnification for the brains. These settings were kept constant for all experiments. No manipulations of brightness and contrast were performed before absolute CRY staining intensity was measured in single confocal pictures of retinas and brains. Staining intensity was measured with ImageJ (FIJI, available at http://fiji.sc/Downloads) in gray-level values within a 9 × 9 pixel area within and outside the stained structures.

In the brains, the 9 × 9 pixel area was laid into each of the four PDF positive large ventrolateral neurons (l-LN_v_s) of one brain hemisphere and into an area close but outside of them in order to monitor background staining. The CRY-staining values of all four l-LN_v_s were averaged for each brain hemisphere and the background subtracted. This was done for 12 brain hemispheres to obtain the average CRY staining at ZT11 and ZT23 of the light-dark cycle.

In the retinas, the 9 × 9 pixel area was laid into the rhabdomeres and into the cytoplasm (soma) of photoreceptor cells R1–6 as well as into inter-rhabdomeric space (background). This was done in four different ommatidia of the same retina, respectively. The values of all ommatidia (rhabdomeres and soma) were averaged and the background subtracted. This measure was repeated for 12 retinas of the different samples (DD, 2hL, ZT11 and ZT23), respectively, and average gray values were obtained for rhabdomeric and cytoplasmic retina staining.

For showing pictures in the figures, image size, brightness and contrast of the pictures were adjusted with GIMP (2.8.6, Kimball and Mattis) and Powerpoint 2010 (Microsoft Office) or Corel Photopaint (CorelDraw Graphics Suite X6, 64 bit).

### Recording of Locomotor Activity Rhythms and Data Analysis

Locomotor activity was recorded under constant temperature (20°C) from 2–6 day old male flies using the custom-made system described in Schlichting and Helfrich-Förster (2015) or the Drosophila Activity Monitors from Trikinetics Incorporation (Waltham, MA, USA). For the white-light experiments, flies were exposed to a 1-week light-dark cycle (LD) of 12 h light and 12 h darkness at either 10, 100, 1000 or 10,000 lux. The average activity profile and the relative nocturnal activity level were calculated as described in Schlichting and Helfrich-Förster (2015). For the red-light/dark experiments, flies were exposed to 12:12 h red-light/dark (RD) cycles (300 μW/cm^2^ red-light during the light phase). After 6 days of recording, the RD cycle was phase-delayed by 8 h. A second phase delay was performed after the flies have phase-shifted their locomotor activity to the first phase delay. Average actograms were calculated from all flies of a given genotype with ActogramJ v0.9 (Schmid et al., 2011) a plugin of Fiji, v1.0 (Schindelin et al., 2012) and the number of days needed for the phase-shift as well as the number of hours phase-shifted after 3 days was determined on individual flies with the help of the Fiji tool “Acrophase” as described in Eck et al. (2016). Briefly, the daily acrophase of the rhythm was plotted into the actogram of each individual fly (see Figure 4) and it was determined manually how long the fly took for re-entrainment and by how many hours it has shifted its acrophase on day 3 after the shift. This was done for both phase shifts separately, and the measured values were averaged for each fly.

**Figure 1:**
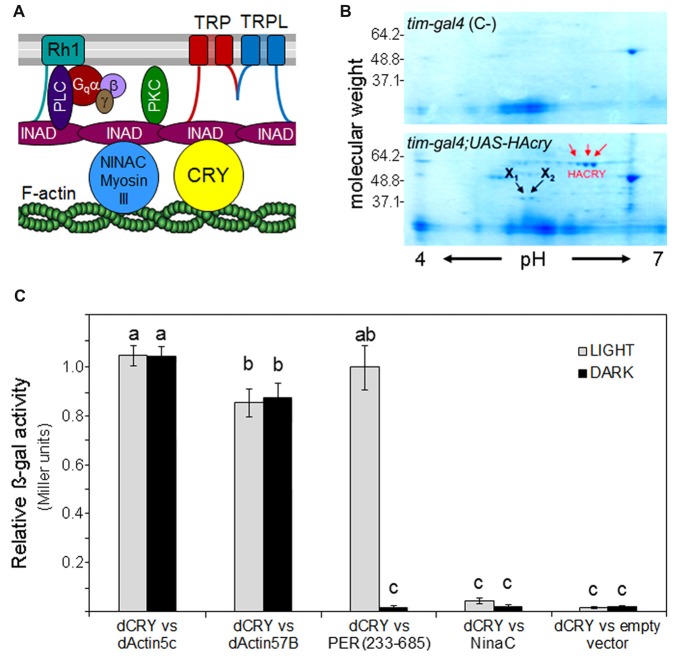
Cryptochrome (CRY) interacts with F-Actin. **(A)** Putative position of CRY in the phototransduction cascade of the fly rhabdomere. The cartoon is modified after Montell (2012). INAD (**I**nactivation **N**o **A**fterpotential**D**) is a crucial PDZ-scaffold protein which gathers together many components of the cascade. It is connected to F-actin via the MyosinIII protein NINAC as well as via CRY (according to the present results). In addition, INAD interacts with rhodopsin 1 (Rh1), the transient-receptor-potential channels TRP and TRPL, Phospholipase C (PLC) and Phosphokinase C (PKC). **(B)** Coomassie blue-stained 2D gel of head protein extracts co-immunoprecipitated with an anti-HA antibody. HACRY overexpressing flies (*yw;tim-gal4/+;uas-HAcry/+*) and relative control (*yw;tim-gal4* (C-)) have been reared in 12:12 LD and collected in the dark (ZT24). Protein complexes have been subjected to 2D separation (1st dimension: IPG STRIP pH 4–7; 2nd dimension NuPage ZOOM gel 4%–12% Invitrogen). Red arrows indicate the spots relative to HACRY, while X1 and X2 are spots corresponding to putative HACRY partners. **(C)** Yeast two-hybrid assays showing the light-independent interaction between dCRY and dAct-5C and dAct-57B. A fragment of PER (aa 233–685), known to interact with dCRY in a light-dependent manner, and NinaC were used as positive and negative control of the interaction, respectively. The activity of the empty prey vector is considered as background. Reported is the β-galactosidase activity (Miller units) normalized to the activity of PER_(233–685)_ in light. Mean ± SEM of seven independent clones, analyzed in triplicates, is shown. For the controls and for the “empty vector”, three clones were tested. Statistics: one-way ANOVA followed by Tukey’s multiple comparisons test. Significantly different values are marked with different letters.

**Figure 2:**
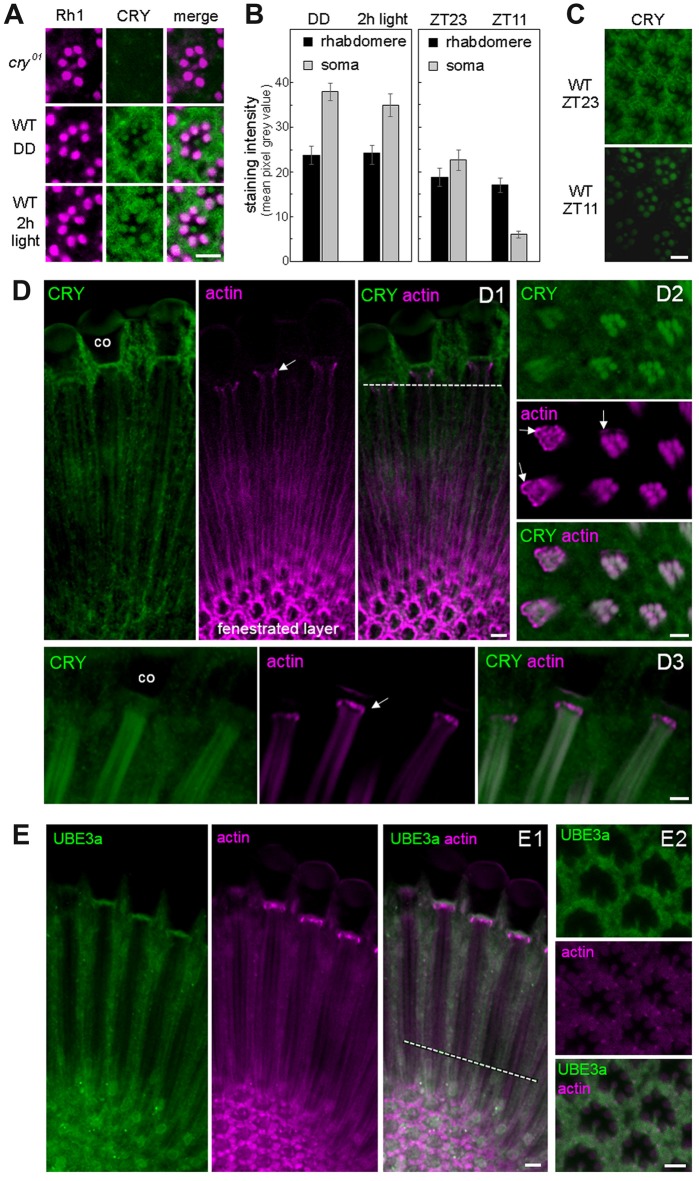
CRY is stably expressed in the rhabdomeres of the photoreceptor cells, co-localizes with actin but only marginally with Ubiquitin Ligase3. **(A)** Cross sections of one ommatidium, respectively, stained with anti-Rh1 (magenta) and anti-CRY (green). No CRY staining is present in *cry*^01^ mutants, whereas in wild-type (WT) flies CRY is detected in all eight photoreceptor cells including their rhabdomeres. After 2 h illumination with 1000 lux, rhabdomeric CRY staining did not disappear. **(B)** Quantification of CRY staining intensity in the rhabdomeres and photoreceptor somata of WT flies raised in constant darkness (DD), after subsequent 2-h exposure to 1000 lux and under a regular 12:12 light-dark cycles at the end of the night (ZT23) and end of the day (ZT11), respectively. Means (± SEM) of 12 independent retinas, respectively, are shown. In the rhabdomeres, CRY-staining was not reduced after 2-h light-exposure (*p* = 0.404) and only slightly at ZT11 during the regular light-dark cycle in comparison to the 2-h light exposure (*p* = 0.001). During the light-dark cycle, CRY staining of the rhabdomeres was the same at ZT23 and ZT11 (*p* = 1.0), but CRY staining in the somata of the photoreceptor cells was much lower at ZT11 than at ZT23 (*p* < 0.001). CRY staining in the somata of the photoreceptor cells was very high after keeping the flies in DD and was not significantly reduced after the 2-h light exposure (*p* = 0.275). **(C)** Examples of retinal CRY staining at ZT23 and ZT11. During the day (ZT11) CRY was significantly lower than during the night (ZT23) in the photoreceptor somata (*p* > 0.0001) but not in the rhabdomeres (*p* = 1.0). Statistics: one-way ANOVA followed by Tukey’s multiple comparisons test. **(D)** Co-localization of CRY and actin (visualized by fluorochrome-conjugated Phalloidin) in the retina in two longitudinal views **(D1,D3)** and one cross section **(D2)**. The position of the cross section is indicated by the broken white line in D1. Actin-Phalloidin staining was variable, but always very strong in the fenestrated layer at the bottom of the retina **(D1)** and in the distal retina just below the cristal cones (co; arrows in **D1–D3**). In the latter place, actin surrounded the photoreceptor cells (arrows in **D2**). To a weaker extent, actin was also always present in the rhabdomeres, where it co-localized with CRY (**D2, D3** and weakly in **D1**). **(E)** Co-localization of ubiquitin ligase 3a (UBE3a) and actin in the retina in a longitudinal **(E1)** view and a cross section **(E2)** at ZT23. The position of the cross section is indicated by the broken white line in **(E1)**. UBE3a was highly expressed in the soma of the photoreceptor cells but only marginally in the rhabdomeres. Scale bars: 5 μm.

**Figure 3:**
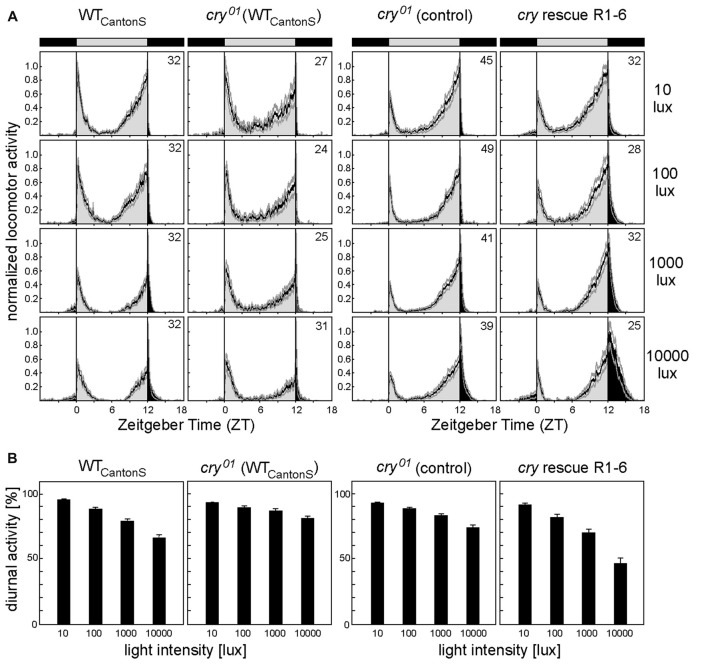
CRY in the compound eyes enhances sensitivity to daylight. **(A)** Average locomotor activity profiles of WT flies (WT_CantonS_), *cry*^01^ mutants (*cry*^01^ (WT_CantonS_)), *cry*^01^ controls and *cry*^01^ mutants with CRY rescued in photoreceptor cells 1–6 (R1–6) under light-dark cycles with different daylight intensities. *cry*^01^ controls consist of ~half *ninaE-gal4;cry*^01^ flies and half *UAS-cry;cry*^01^ flies, respectively. We pooled the two controls, because they behaved similarly (*p* = 0.176). Flies were recorded under light-dark cycles with 12 h of light and 12 h of darkness (LD 12:12) with daylight intensities of 10, 100, 1000 and 10,000 lux, respectively. Light period (bar on top) and activity during the day are shown in light gray whereas the dark period (bar on top) and activity during the night are shown in black. Average activity profiles are normalized with maximal activity set to one. Faint gray lines above and below the average profiles represent standard errors of the mean (+SEM). Number of recorded animals are given in the right top corner of the upper diagram. **(B)** Mean percentage diurnal activity of total daily activity (± SEM) calculated for WT flies (WT_CantonS_), *cry*^01^ mutants (*cry*^01^ (WT_CantonS_) and *cry*^01^ controls) and *cry*^01^ mutants with CRY rescued in photoreceptor cells 1–6 (R1–6) under light-dark cycles with different daylight intensities. A two-way ANOVA showed that relative diurnal activity depended significantly on daylight intensity (*F*_(3,504)_ = 172.017; *p* < 0.001) and on the strain (*F*_(3,5204)_ = 53.108; *p* < 0.001) and that there was a significant interaction between the two (*F*_(9,522)_ = 11.882; *p* < 0.001), indicating that diurnal activity decreased differently with increasing daylight intensity in the different strains. *Post hoc* analysis revealed significant differences between *cry*^01^ (WT_CantonS_) mutants and WT_CantonS_ flies (*p* < 0.0001) as well as between *cry*^01^ controls (*ninaE-gal4;cry*^01^ and *uas-cry;cry*^01^ pooled) and *cry*^01^ mutants with CRY rescued in photoreceptor cells R1–6 (*ninaE-gal4;uas-cry;cry*^01^; *p* < 0.0001). Statistics: two-way ANOVA followed by Tukey’s multiple comparisons test.

**Figure 4:**
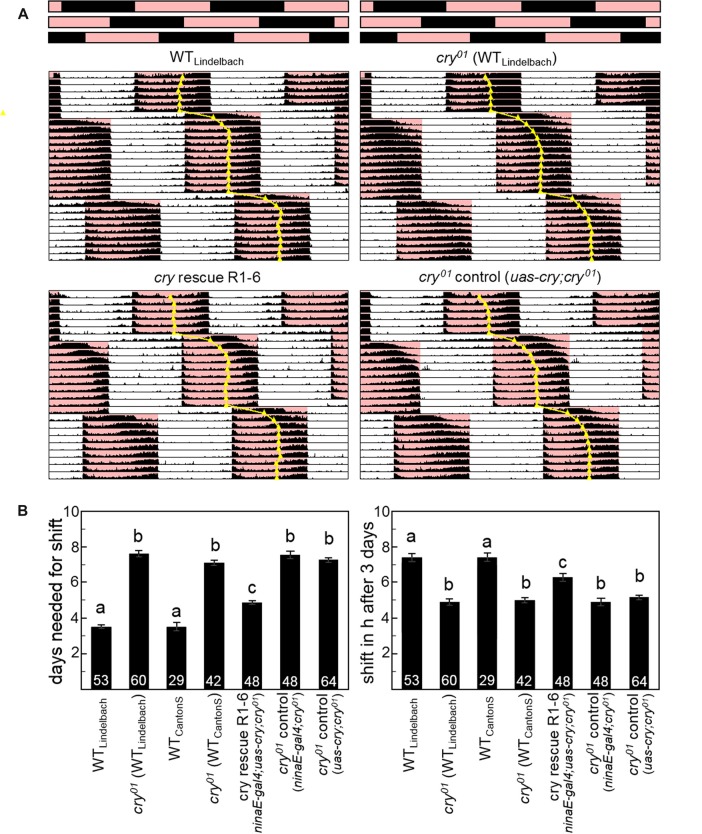
CRY is essential for a fast re-entrainment of the activity rhythms to delays of 12:12 red-dark cycles (RD). **(A)** Average actograms of WT flies (*n* = 53), *cry*^01^ mutants (*n* = 60; both in the WT “Lindelbach” genetic background) and *cry*^01^ mutants with cry rescued in photoreceptors R1–6 (*n* = 48; *ninaE-gal4;uas-cry;cry*^01^) plus relevant controls (*n* = 64; *uas-cry;cry*^01^). Bars on top represent the RD cycle (R = red, D = black) that is phase-delayed by 8 h two times during the recording. The daily median of activity is indicated, as judgment of rhythm phase. **(B)** Quantification of the phase shifts. The left diagram shows the number of days flies of different genotypes needed to re-entrain to the phase-delayed RD cycle (means ± SEM). The right diagram depicts the number of hours the flies had phase-shifted at day 3 (means ± SEM). Numbers in columns indicate the number of tested flies. Strains that are significantly different from each other (*p* < 0.05) are marked by different letters.

### Statistical Analysis

Statistical analysis was performed with Systat11 or Graphpad Prism v4. After checking for normal distribution, data were compared by either a one- or two-way ANOVA followed by a pairwise comparison or Tukey’s multiple comparisons test if normality was retained. If normality was rejected a Mann-Whitney-U or Wilcoxon-test were applied.

## Results and Discussion

### CRY Interacts With F-actin

F-actin, one of the major cytoskeletal components, is highly expressed in the rhabdomeric microvilli of fly photoreceptors and helps maintaining their structure (Arikawa et al., 1990). In addition, F-actin seems to be involved in subcellular localization and functional coupling of the phototransduction components, putatively via interaction with the myosin III protein NINAC (Figure 1A; Lee and Montell, 2004). NINAC also interacts with the scaffolding PDZ-domain protein INAD (Figure 1A), possibly contributing to movements of phototransduction components into or out of the rhabdomeres and hence interfering with photosensitivity and light-adaptation (reviewed in Montell, 2012). However, no such role of NINAC was found so far, as its main function seems to lie in the inactivation of Metarhodopsin by accelerating the binding of Arrestin (Liu et al., 2008). Here, we asked whether CRY, which was also found to interact with INAD (Mazzotta et al., 2013), could cover this function.

A preliminary screening of an adult head cDNA library led to the identification of dActin-57B as putative CRY partner. Then, a Co-IP assay, followed by 2D electrophoresis and mass spectrometry analysis, was performed on transgenic fly heads overexpressing a hemagglutinin (HA)-tagged form of CRY (HACRY) in all clock and photoreceptor cells by the use of a *tim-gal4* driver (Dissel et al., 2004). Flies were raised in 12:12 light:dark cycles and collected before lights-on and HACRY was pulled-down using a HA-affinity matrix (See Supplementary Materials and Methods). Two spots of ~40 kDa (X1 and X2 in Figure 1B) were observed in the sample but absent in the negative control (Figure 1B). These protein bands were digested in-gel and the peptide mixtures were analyzed by liquid chromatography–tandem mass spectrometry (LC-MS/MS; Wilm et al., 1996). Analysis of the LC-MS/MS data using the MASCOT software yielded the identification of dActin-87E and dActin-5C in spot X1 and of dActin-57B in spot X2 (Supplementary Table S3). In *Drosophila melanogaster*, the actin family comprises six members: four of them, Act88F, Act79B, Act57B and Act87E, are muscle specific actins, while Act5C and Act42A are cytoplasmic. These two isoforms, ubiquitously expressed during all developmental stages, differ for only two amino acids (Supplementary Figure S2). Our MS results from adult head extracts are consistent with the tissue expression of the actin isoforms. Actin5C has been identified as a key molecule in the formation of the luminal matrix (= inter-rhabdomeric space), fundamental for shaping and positioning of rhabdomeres for proper visual sensitivity (Nie et al., 2014). Actin57B, the major species in larval intersegmental muscles, is expressed also in adult cephalic and abdominal muscles (Fyrberg et al., 1998), and is a key organizer in the assembly of presynaptic active zone (AZ) that coordinates the synaptic release machinery to facilitate neuronal communication (Blunk et al., 2014).

We decided to further investigate the interactions found by library screening and CoIP using the yeast two-hybrid assay, where a full-length dCRY, directly fused to LexA (bait), has been challenged with the full-length dActin-5C or dActin-57B, as prey. A fragment of PER, aa 233–685, was used as positive control of the interaction: this fragment includes the major protein/protein interaction domains of PER and it is known to interact with dCRY only in presence of light (Rosato et al., 2001; Hemsley et al., 2007). NINAC, that we had previously shown to bind dCRY only in presence of INAD acting as bridge (Mazzotta et al., 2013), was used as negative control. The yeast assay was performed both in the light and in the dark, and a strong light-independent affinity between dCRY and both actins was observed (Figure 1C).

### CRY Is Expressed in the Rhabdomeres of the Photoreceptor Cells and Remains Stable After Photo Activation

Our data suggest that CRY is bound to F-actin during light and darkness and could consequently stabilize INAD also after prolonged illumination. This hypothesis requires CRY to remain stably present under light, which is in contrast to previous observations showing a quick degradation of CRY after light onset in photoreceptor cells, clock neurons and S2 cells (Emery et al., 1998; Koh et al., 2006; Peschel et al., 2006, 2009; Yoshii et al., 2008; Ozturk et al., 2011, 2013). To test the presence of CRY in the rhabdomeres, we immunostained retinas of flies kept in complete darkness from egg hatching onward as well as retinas of flies initially raised under the same conditions but then exposed for 2 h to bright light (1000 lux). CRY immunostaining was not visible in *cry*^01^ mutants (Supplementary Figure S3) but present in all rhabdomeres of WT flies (Figure 2A, Supplementary Figure S4). No sign of CRY degradation could be detected in the rhabdomeres after 2 h of light exposure (Figures 2A,B; Supplementary Figure S4). Very similar, no reduction in rhabdomeric CRY staining could be detected during the light phase of a regular light-dark cycle (Figures 2B,C), whereas cytoplasmic CRY staining in the somata of the photoreceptor cells was strongly reduced during the light phase (=ZT11; Figures 2B,C; Supplementary Figure S5). The latter coincides with the strong reduction of CRY-staining in Western-blots during the light phase (Emery et al., 1998). Also, in the PDF positive l-LN_v_ clock neurons, CRY staining was extremely low at ZT11 in the light phase, whereas it was high at ZT23 during the dark phase (Supplementary Figures S6, S7). In comparison to CRY staining intensity in the clock neurons that reached a mean pixel gray value of 80 during the night (Supplementary Figure S6), CRY staining intensity in the retina was much weaker. In the cytoplasm, it reached maximally a mean pixel gray value of 40 after the flies had been kept in prolonged darkness and a maximal value of 25 during the night of a regular light-dark cycle (Figure 2B). In the rhabdomeres, CRY was even lower and did not exceed a mean pixel gray value of 25, even not after prolonged darkness. This explains why CRY was so far not detected in the rhabdomeres and in the cytoplasm of the photoreceptors cells only after prolonged darkness (Yoshii et al., 2008) or on Western-blots during the dark phase (Emery et al., 1998). Our findings indicate that CRY is stably bound to rhabdomeric F-actin, which may prevent its degradation in the proteasome during the light phase. In order to test the co-localization of CRY and actin, we performed double-staining with anti-CRY and fluorochrome-conjugated Phalloidin that binds selectively to F-actin (Cooper, 1987). We found actin-Phalloidin staining quite variable: in the rhabdomeres the staining was sometimes weak (Figure 2D1), sometimes strong (Figures 2D2,D3) and sometimes it was present also in the cytoplasm of the photoreceptor cells (Figure 2E1). Nevertheless, actin was always present in the rhabdomeres, where it nicely co-localized with CRY (Figure 2D). It is unknown whether the light-activated E3 ligase complex, essential for light-mediated CRY degradation, is present in the rhabdomeres, but it is known that the same E3 ligase complex components that induce CRY ubiquitination, such as the BRWD3 protein Ramshackle and the Cullin4-RING Finger E3 Ligase (CRL4) are associated with chromatin and the nucleus (D’Costa et al., 2006; Jackson and Xiong, 2009; Ozturk et al., 2013). To test whether proteins of the E3 ligase complex are present in the rhabdomeres of the photoreceptor cells, we stained retinas with an antibody against the ubiquitin E3 ligase, UBE3A, which has previously shown to be expressed in the fly compound eyes (Ramirez et al., 2015). We found strong UBE3A staining in the cytoplasm of the photoreceptor cells, but only very weak staining in the rhabdomeres, suggesting that UBE3A is mainly expressed in the cytoplasm (Figure 2E2). This was true at ZT11 and ZT23.

### CRY in the Compound Eyes Contributes to Measuring Daylight Intensity and Adapting Fly Diurnal/Nocturnal Activity Levels

After having shown that CRY interacts with actin and is stably present in the rhabdomeres of the compound eyes, we wanted to test whether this has any biological meaning for the flies, in addition to the already shown small visual impairments of *cry*^01^ mutants (Mazzotta et al., 2013). The fly circadian clock is known to be very sensitive to light, and, therefore, it is an ideal system to study possible influences of CRY (Hirsh et al., 2010; Vanin et al., 2012; Vinayak et al., 2013). CRY is one of the major light-input pathways to the clock neurons (Ozkaya and Rosato, 2012; Zheng and Sehgal, 2012), but its role in the compound eyes for circadian entrainment is so far not understood. The compound eyes seem not important for fast clock responses to light (Stanewsky et al., 1998; Yang et al., 1998; Emery et al., 2000; Kistenpfennig et al., 2012), but they rather appear to fine-tune fly daily activity to different light-conditions (Schlichting et al., 2015) and to set the diurnal/nocturnal activity level ratio (Bachleitner et al., 2007; Rieger et al., 2007). We could recently show that photoreceptors R1–6 are responsible for measuring daylight intensity and for reducing the amount of diurnal activity with increasing daylight intensity (Schlichting et al., 2014). Most importantly, the reduction of diurnal activity with increasing light intensity is independent of a functional clock and can also be observed in *per*^01^ mutants (Kempinger et al., 2009). Thus, this behavior appears suited to measure the light-sensitivity of photoreceptors R1–6 during the day in an easy way.

If CRY in R1–6 contributes to measuring daylight intensity, one would expect a slightly different diurnal/nocturnal activity level ratio in *cry*^01^ mutants. In order to test this hypothesis, we recorded diurnal/nocturnal activity of *cry*^01^ mutants under 12:12 light-dark (LD) cycles of different daylight intensities (10, 100, 1000 and 10,000 lux) and determined the percentage of diurnal activity from whole-day activity (Figure 3A). We found that the decrease in relative diurnal activity with increasing daylight intensity was significantly stronger in WT flies than in *cry*^01^ mutants. To ensure that CRY in the compound eyes is responsible for the observed differences, we expressed CRY under control of the rhodopsin1 promoter (*ninaE*) only in R1–6 in an otherwise *cry*^01^ background. We found that such flies behaved in a WT-like manner. They even decreased diurnal activity with increasing light intensity slightly more than WT flies (Figure 3B) what might be due to different genetic backgrounds of the tested flies. Our results indicate that CRY in R1–6 is indeed involved in measuring daylight intensity, probably by interfering with phototransduction in these photoreceptor cells.

### CRY Action in the Compound Eyes Does Not Depend on Light-Activated CRY

WT flies can well entrain to 12:12 red-dark (RD) cycles and this ability depends on Rh1 and Rh6 in the compound eyes while it is independent of CRY (Helfrich-Förster et al., 2002; Hanai et al., 2008). If our hypothesis is right and CRY keeps the components of the transduction cascade close to the photoreceptor membrane, the entrainability of the flies to red light should be reduced when CRY is absent. To test this, we compared the ability of WT flies and *cry*^01^ mutants (in two different backgrounds) to follow an 8-h delay of a 12:12 RD cycle (Figure 4; Supplementary Figure S8). We found that both WT strains needed about 3–4 days to accomplish the shift, whereas the two *cry*^01^ mutants took about 7 days to do so. This difference is highly significant (*p* < 0.001) and is a strong indication for a role of CRY in Rhodopsin-based light transduction that is independent of CRY’s own photoreceptive capabilities. In order to test whether this role of CRY takes indeed place in the compound eyes, we expressed *cry* in the Rh1 expressing photoreceptors R1–6 of *cry*^01^ mutants. Indeed, we could partially rescue the WT speed to re-entrain to a shifted RD cycle: *ninaE-gal4;uas-cry;cry*^0^ flies took 5 instead of 3–4 days to re-entrain completely (Figure 4; Supplementary Figure S9). A reason for this partial rescue might be the lack of CRY in the Rh6 containing inner photoreceptor cells that also contribute to red light entrainment (Hanai et al., 2008).

### Human CRY2 Appears to Interact Also With Human Actin Beta in a Light-Independent Manner

**Figure 5:**
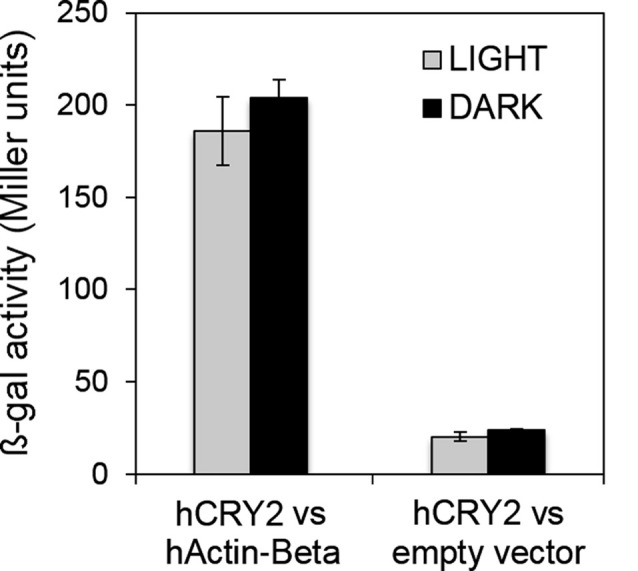
Human CRY2 also interacts with actin. Yeast two-hybrid assays showing the light-independent interaction between hCRY2 and hActin-Beta. β-galactosidase activity (Miller units) is reported. Mean ± SEM of seven independent clones, analyzed in triplicates, is shown. For the “empty vector”, three clones were tested. Statistics: t-Student.

Although vertebrate CRYs act as transcriptional regulators in the circadian clock, they have been suggested to influence the sensitivity of the pupillary light response in mammals, in a fashion that is independent from a role as photopigment (Owens et al., 2012). Humans possess two CRYs of type 2, both expressed in the retina. CRY2 mRNA seems to be much more abundant than CRY1 mRNA in the adult retina and CRY2 protein has been detected in most cells in the ganglion cell layer (GCL) and in a subset of cells in the inner nuclear layer (INL; Thompson et al., 2003). In a very preliminary and purely indicative yeast two-hybrid assay, we have challenged the full-length hCRY2, directly fused to LexA (bait), with the full-length human Actin-beta (hActin-Beta; reported to be one of the two non-muscle cytoskeletal actins; RefSeq, NCBI Reference Sequence Database) as prey. The assay, performed both in light and dark, has revealed a light-independent interaction between the two proteins (Figure 5). This suggests that also mammalian CRYs could anchor to the actin cytoskeleton, raising the hypothesis that they could stabilize the phototransduction complex to the membrane of the intrinsically photosensitive retinal ganglion cells (ipRGCs), therefore contributing to the inner retinal photoreception.

## Conclusion

We had previously uncovered a role for CRY in fly visual biology, by the interaction with the phototransduction cascade (Mazzotta et al., 2013). Here, we show that through this interaction it also slightly increases light-sensitivity of the eyes, and WT flies may sense day-light, nocturnal light and red light as being brighter than *cry*^01^ mutants do. This role of CRY in the fly retina is rather independent of its function as photopigment, as CRY seems to act as a stabilizing protein keeping the INAD signalplex linked to the F-actin and therefore to the rhabdomere internal membrane.

This non-photoreceptive role of CRY in the retina could be a feature shared with mammals. In fact, mammalian CRYs are expressed in the retina, especially in the ganglion cells responsible for circadian entrainment and pupillary responses (Thresher et al., 1998; Thompson et al., 2003). Nowadays, it is clear that melanopsin—not CRYs—in the retinal ganglion cells is the major mammalian circadian photopigment (Hattar et al., 2002; Panda et al., 2002; Ruby et al., 2002; Lucas et al., 2003; Peirson and Foster, 2006). Nevertheless, several reports suggest that CRYs affect circadian photoreception and pupillary responses (Miyamoto and Sancar, 1998; Selby et al., 2000; Van Gelder et al., 2003). Also, mammalian CRYs could stabilize the phototransduction complex at the membrane of retinal cells. This is conceivable because melanopsin ganglion cells have an insect-like (rhabdomeric) phototransduction cascade employing Gq/11-class G proteins and Phospholipase C (PLC; Graham et al., 2008). This tempting hypothesis is reinforced by our finding that human CRY2 is able to interact with human Actin-Beta in a light-independent manner.

The role for CRY we propose here is new and clearly different from the recently shown CRY action at the membrane of the large lateral ventral neurons, where light-activated CRY evokes rapid membrane depolarization through the redox sensor of the voltage-gated ß-subunit potassium channel hyperkinetic (Fogle et al., 2015). Though, we cannot completely exclude such a role for CRY in the photoreceptor cells, our results rather speak for a role of CRY in stabilizing the signalplex components at the rhabdomeres.

## Author Contributions

CH-F, GM and RC conceived and supervised the study. MS performed and analyzed the behavioral experiments with white light. DR performed and analyzed those with red light. RG, MS and CH-F did the immunostaining in the compound eyes and the brain. PC and GM performed the co-immunoprecipitation assays and the yeast two-hybrid experiments. CH-F, GM and RC wrote the manuscript. Correspondence should be addressed to CH-F or GM.

## Conflict of Interest Statement

The authors declare that the research was conducted in the absence of any commercial or financial relationships that could be construed as a potential conflict of interest.

## Footnotes

^1^http://www.matrixscience.com

## References

[B1] Arikawa K. Hicks J. L. Williams D. S. (1990). Identification of actin filaments in the rhabdomeral microvilli of *Drosophila* photoreceptors. J. Cell Biol. 110, 1993–1998. 10.1083/jcb.110.6.1993 2112548 PMC2116135

[B2] Ausbel F. M. (1998). Current Protocols in Molecular Biology. New York, NY: Green Publishing Associated.

[B3] Bachleitner W. Kempinger L. Wülbeck C. Rieger D. Helfrich-Förster C. (2007). Moonlight shifts the endogenous clock of *Drosophila melanogaster*. Proc. Natl. Acad. Sci. U S A 104, 3538–3543. 10.1073/pnas.0606870104 17307880 PMC1805525

[B5] Biscontin A. Wallach T. Sales G. Grudziecki A. Janke L. Sartori E.. (2017). Functional characterization of the circadian clock in the Antarctic krill, Euphausia superba. Sci. Rep. 7:17742. 10.1038/s41598-017-18009-2 29255161 PMC5735174

[B6] Blunk A. D. Akbergenova Y. Cho R. W. Lee J. Walldorf U. Xu K.. (2014). Postsynaptic actin regulates active zone spacing and glutamate receptor apposition at the *Drosophila* neuromuscular junction. Mol. Cell. Neurosci. 2014, 241–254. 10.1016/j.mcn.2014.07.005 25066865 PMC4134997

[B7] Ceriani M. F. Darlington T. K. Staknis D. Más P. Petti A. A. Weitz C. J.. (1999). Light-dependent sequestration of TIMELESS by CRYPTOCHROME. Science 285, 553–556. 10.1126/science.285.5427.553 10417378

[B8] Chaves I. Pokorny R. Byrdin M. Hoang N. Ritz T. Brettel K.. (2011). The cryptochromes: blue light photoreceptors in plants and animals. Annu. Rev. Plant Biol. 62, 335–364. 10.1146/annurev-arplant-042110-103759 21526969

[B9] Collins B. H. Rosato E. Kyriacou C. P. (2004). Seasonal behavior in *Drosophila melanogaster* requires the photoreceptors, the circadian clock and phospholipase C. Proc. Natl. Acad. Sci. U S A 101, 1945–1950. 10.1073/pnas.0308240100 14766972 PMC357032

[B10] Cooper J. A. (1987). Effects of cytochalasin and phalloidin on actin. J. Cell Biol. 105, 1473–1478. 10.1083/jcb.105.4.1473 3312229 PMC2114638

[B11] Damulewicz M. Mazzotta G. M. Sartori E. Rosato E. Costa R. Pyza E. (2017). Cryptochrome is a regulator of synaptic plasticity in the visual system of *Drosophila melanogaster*. Front. Mol. Neurosci. 10:165. 10.3389/fnmol.2017.00165 28611590 PMC5448152

[B12] D’Costa A. Reifegerste R. Sierra S. Moses K. (2006). The *Drosophila* ramshackle gene encodes a chromatin-associated protein required for cell morphology in the developing eye. Mech. Dev. 123, 591–604. 10.1016/j.mod.2006.06.007 16904300

[B13] Dissel S. Codd V. Fedic R. Garner K. J. Costa R. Kyriacou C. P.. (2004). A constitutively active cryptochrome in *Drosophila melanogaster*. Nat. Neurosci. 7, 834–840. 10.1038/nn1285 15258584

[B14] Dolezelova E. Dolezel D. Hall J. C. (2007). Rhythm defects caused by newly engineered null mutations in *Drosophila’s* cryptochrome gene. Genetics 177, 329–345. 10.1534/genetics.107.076513 17720919 PMC2013679

[B15] Eck S. Helfrich-Förster C. Rieger D. (2016). The timed depolarizaiton of morning and evening oscillators phase shifts the circadian clock of *Drosophila*. J. Biol. Rhythms 31, 428–442. 10.1177/0748730416651363 27269519

[B16] Emery P. So W. V. Kaneko M. Hall J. C. Rosbash M. (1998). CRY, a *Drosophila* clock and light-regulated cryptochrome, is a major contributor to circadian rhythm resetting and photosensitivity. Cell 95, 669–679. 10.1016/s0092-8674(00)81637-2 9845369

[B17] Emery P. Stanewsky R. Helfrich-Förster C. Emery-Le M. Hall J. C. Rosbash M. (2000). *Drosophila* CRY is a deep brain circadian photoreceptor. Neuron 26, 493–504. 10.1016/s0896-6273(00)81181-2 10839367

[B18] Fedele G. Edwards M. D. Bhutani S. Hares J. M. Murbach M. Green E. W.. (2014). Genetic analysis of circadian responses to low frequency electromagnetic fields in *Drosophila melanogaster*. PLoS Genet. 10:e1004804. 10.1371/journal.pgen.1004804 25473952 PMC4256086

[B19] Fogle K. J. Baik L. S. Houl J. H. Tran T. T. Roberts L. Dahm N. A.. (2015). CRYPTOCHROME-mediated phototransduction by modulation of the potassium ion channel ß-subunit redox sensor. Proc. Natl. Acad. Sci. U S A 112, 2245–2250. 10.1073/pnas.1416586112 25646452 PMC4343116

[B20] Fogle K. J. Parson K. G. Dahm N. A. Holmes T. C. (2011). CRYPTOCHROME is a blue-light sensor that regulates neuronal firing rate. Science 331, 1409–1413. 10.1126/science.1199702 21385718 PMC4418525

[B21] Foley L. E. Gegear R. J. Reppert S. M. (2011). Human cryptochrome exhibits light-dependent magnetosensitivity. Nat. Commum. 2:356. 10.1038/ncomms1364 21694704 PMC3128388

[B22] Fyrberg E. A. Fyrberg C. C. Biggs J. R. Saville D. Beall C. J. Ketchum A. (1998). Functional nonequivalence of *Drosophila* actin isoforms. Biochem. Genet. 36, 271–287. 10.1023/A:1018785127079 9791722

[B23] Gegear R. J. Foley L. E. Casselman A. Reppert S. M. (2010). Animal cryptochromes mediate magnetoreception by an unconventional photochemical mechanism. Nature 463, 804–807. 10.1038/nature08719 20098414 PMC2820607

[B24] Golemis E. A. Brent R. (1997). “Searching for interacting proteins with the two-hybrid system III,” in The Yeast Two-Hybrid System, eds Bartel P. L. Field S. (New York, NY: Oxford University Press), 43–72.

[B25] Golombek D. A. Rosenstein R. E. (2010). Physiology of circadian entrainment. Physiol. Rev. 90, 1063–1102. 10.1152/physrev.00009.2009 20664079

[B26] Graham D. M. Wong K. Y. Shapiro P. Frederick C. Pattabiraman K. Berson D. M. (2008). Melanopsin ganglion cells use a membrane-associated rhabdomeric phototransduction cascade. J. Neurophysiol. 99, 2522–2532. 10.1152/jn.01066.2007 18305089

[B27] Griffin E. A. Jr. Staknis D. Weitz C. J. (1999). Light-independent role of CRY1 and CRY2 in the mammalian circadian clock. Science 286, 768–771. 10.1126/science.286.5440.768 10531061

[B28] Hanai S. Hamasaka Y. Ishida N. (2008). Circadian entrainment to red light in *Drosophila*, requirement of Rhodopsin 1 and Rhodopsin 6. Neuroreport 19, 1441–1444. 10.1097/WNR.0b013e32830e4961 18766027

[B29] Hattar S. Liao H. W. Takao M. Berson D. M. Yau K. W. (2002). Melanopsin-containing retinal ganglion cells, architecture, projections, and intrinsic photosensitivity. Science 295, 1065–1070. 10.1126/science.1069609 11834834 PMC2885915

[B30] Helfrich-Förster C. Edwards T. Yasuyama K. Wisotzki B. Schneuwly S. Stanewsky R.. (2002). The extraretinal eyelet of *Drosophila*, Development, ultrastructure, and putative circadian function. J. Neurosci. 22, 9255–9266. 10.1523/JNEUROSCI.22-21-09255.2002 12417651 PMC6758046

[B31] Hemsley M. J. Mazzotta G. M. Mason M. Dissel S. Toppo S. Pagano M. A.. (2007). Linear motifs in the C-terminus of *D. melanogaster* cryptochrome. Biochem. Biophys. Res. Commun. 355, 531–537. 10.1016/j.bbrc.2007.01.189 17306225

[B32] Hirsh J. Riemensperger T. Coulom H. Iché M. Coupar J. Birman S. (2010). Roles of dopamine in circadian rhythmicity and extreme light sensitivity of circadian entrainment. Curr. Biol. 20, 209–214. 10.1016/j.cub.2009.11.037 20096587 PMC2811851

[B33] Hoang N. Schleicher E. Kacprzak S. Bouly J. P. Picot M. Wu W.. (2008). Human and *Drosophila* cryptochromes are light activated by flavin photoreduction in living cells. PLoS Biol. 6:e160. 10.1371/journal.pbio.0060160 18597555 PMC2443192

[B34] Hsiao H. Y. Johnston R. J. Jr. Jukam D. Vasiliauskas D. Desplan C. Rister J. (2012). Dissection and immunohistochemistry of larval, pupal and adult *Drosophila* retinas. J. Vis. Exp. 69:e4347. 10.3791/4347 23183823 PMC3523422

[B35] Ivanchenko M. Stanewsky R. Giebultowicz J. M. (2001). Circadian photoreception in *Drosophila*, functions of cryptochrome in peripheral and central clocks. J. Biol. Rhythms 16, 205–215. 10.1177/074873001129001917 11407780

[B36] Jackson S. Xiong Y. (2009). CRL4s: the CUL4-RING E3 ubiquitin ligases. Trends Biochem. Sci. 34, 562–570. 10.1016/j.tibs.2009.07.002 19818632 PMC2783741

[B37] Johnsson A. Helfrich-Förster C. Engelmann W. (2015). “How light resets circadian clocks,” in Photobiology, ed. Bjorn L. O. (Berlin, Germany: Springer Verlag), 293–297.

[B38] Kempinger L. Dittmann R. Rieger D. Helfrich-Förster C. (2009). The nocturnal activity of fruit flies exposed to moonlight is partly caused by direct light-effects on the activity level that bypass the endogenous clock. Chronobiol. Int. 26, 151–166. 10.1080/07420520902747124 19212834

[B39] Khoudoli G. A. Porter I. M. Blow J. J. Swedlow J. R. (2004). Optimisation of the two-dimensional gel electrophoresis protocol using the Taguchi approach. Proteome Sci. 2:6. 10.1186/1477-5956-2-6 15357868 PMC517948

[B40] Kistenpfennig C. Hirsh J. Yoshii T. Helfrich-Förster C. (2012). Phase-shifting the fruit fly clock without cryptochrome. J. Biol. Rhythms 27, 117–125. 10.1177/0748730411434390 22476772

[B41] Koh K. Zheng X. Sehgal A. (2006). JETLAG resets the *Drosophila* circadian clock by promoting light-induced degradation of TIMELESS. Science 312, 1809–1812. 10.1126/science.1124951 16794082 PMC2767177

[B42] Krishnan B. Levine J. D. Lynch M. K. Dowse H. B. Funes P. Hall J. C. (2001). A new role for cryptochrome in a *Drosophila* circadian oscillator. Nature 411, 313–317. 10.1038/35077094 11357134

[B43] Kume K. Zylka M. J. Sriram S. Shearman L. P. Weaver D. R. Jin X.. (1999). mCRY1 and mCRY2 are essential components of the negative limb of the circadian clock feedback loop. Cell 98, 193–205. 10.1016/s0092-8674(00)81014-4 10428031

[B44] Lee S. J. Montell C. (2004). Light-dependent translocation of visual arrestin regulated by the NINAC myosin III. Neuron 43, 95–103. 10.1016/j.neuron.2004.06.014 15233920

[B45] Liu C. H. Satoh A. K. Postma M. Huang J. Ready D. F. Hardie R. C. (2008). Ca^2+^-dependent metarhodopsin inactivation mediated by calmodulin and NINAC myosin III. Neuron 59, 778–789. 10.1016/j.neuron.2008.07.007 18786361 PMC2562427

[B46] Lu Y. Wang F. Li Y. Ferris J. Lee J.-A. Gao F.-B. (2009). The *Drosophila* homologue fo the Angelman syndrome ubiquitin ligase regulates the formation of terminal dendritic branches. Hum. Mol. Genet. 18, 454–462. 10.1093/hmg/ddn373 18996915 PMC2638802

[B47] Lucas R. J. Hattar S. Takao M. Berson D. M. Foster R. G. Yau K. W. (2003). Diminished pupillary light reflex at high irradiances in melanopsin-knockout mice. Science 299, 245–247. 10.1126/science.1077293 12522249

[B48] Mazzotta G. Rossi A. Leonardi E. Mason M. Bertolucci C. Caccin L.. (2013). Fly cryptochrome and the visual system. Proc. Natl. Acad. Sci. U S A 110, 6163–6168. 10.1073/pnas.1212317110 23536301 PMC3625353

[B49] Mineki R. Taka H. Fujimura T. Kikkawa M. Shindo N. Murayama K. (2002). *In situ* alkylation with acrylamide for identification of cysteinyl residues in proteins during one- and two-dimensional sodium dodecyl sulphate-polyacrylamide gel electrophoresis. Proteomics 2, 1672–1681. 10.1002/1615-9861(200212)2:12<1672::aid-prot1672>3.0.co;2-# 12469337

[B50] Miyamoto Y. Sancar A. (1998). Vitamin B2-based blue-light photoreceptors in the retinohypothalamic tract as the photoactive pigments for setting the circadian clock in mammals. Proc. Natl. Acad. Sci. U S A 95, 6097–6102. 10.1073/pnas.95.11.6097 9600923 PMC27591

[B51] Montell C. (1999). Visual transduction in *Drosophila*. Annu. Rev. Cell Dev. Biol. 15, 231–268. 10.1146/annurev.cellbio.15.1.231 10611962

[B52] Montell C. (2007). Dynamic regulation of the INAD signaling scaffold becomes crystal clear. Cell 131, 19–21. 10.1016/j.cell.2007.09.022 17923082

[B53] Montell C. (2012). *Drosophila* visual transduction. Trends Neurosci. 35, 356–363. 10.1016/j.tins.2012.03.004 22498302 PMC3367115

[B54] Nie J. Mahato S. Zelhof A. C. (2014). The actomyosin machinery is required for *Drosophila* retinal lumen formation. PLoS Genet. 10:e1004608. 10.1371/journal.pgen.1004608 25233220 PMC4168998

[B55] Owens L. Buhr E. Tu D. C. Lamprecht T. L. Lee J. Van Gelder R. N. (2012). Effect of circadian clock gene mutations on nonvisual photoreception in the mouse. Invest. Ophthalmol. Vis. Sci. 53, 454–460. 10.1167/iovs.11-8717 22159024 PMC3292377

[B56] Ozgur S. Sancar A. (2003). Purification and properties of human blue-light photoreceptor cryptochrome 2. Biochemistry 42, 2926–2932. 10.1021/bi026963n 12627958

[B57] Ozkaya O. Rosato E. (2012). The circadian clock of the fly: a neurogenetics journey through time. Adv. Genet. 77, 79–123. 10.1016/b978-0-12-387687-4.00004-0 22902127

[B58] Ozturk N. Selby C. P. Annayev Y. Zhong D. Sancar A. (2011). Reaction mechanism of *Drosophila* cryptochrome. Proc. Natl. Acad. Sci. U S A 108, 516–521. 10.1073/pnas.1017093108 21187431 PMC3021015

[B59] Ozturk N. VanVickle-Chavez S. J. Akileswaran L. Van Gelder R. N. Sancar A. (2013). Ramshackle (Brwd3) promotes light-induced ubiquitylation of *Drosophila* Cryptochrome by DDB1-CUL4-ROC1 E3 ligase complex. Proc. Natl. Acad. Sci. U S A 110, 4980–4985. 10.1073/pnas.1303234110 23479607 PMC3612607

[B60] Panda S. Sato T. K. Castrucci A. M. Rollag M. D. DeGrip W. J. Hogenesch J. B.. (2002). Melanopsin (Opn4) requirement for normal light-induced circadian phase shifting. Science 298, 2213–2216. 10.1126/science.1076848 12481141

[B61] Peirson S. Foster R. G. (2006). Melanopsin: another way of signaling light. Neuron 49, 331–339. 10.1016/j.neuron.2006.01.006 16446137

[B62] Peschel N. Chen K. F. Szabo G. Stanewsky R. (2009). Light-dependent interactions between the *Drosophila* circadian clock factors cryptochrome, jetlag, and timeless. Curr. Biol. 19, 241–247. 10.1016/j.cub.2008.12.042 19185492

[B63] Peschel N. Veleri S. Stanewsky R. (2006). Veela defines a molecular link between Cryptochrome and Timeless in the light-input pathway to *Drosophila’s* circadian clock. Proc. Natl. Acad. Sci. U S A 103, 17313–17318. 10.1073/pnas.0606675103 17068124 PMC1859927

[B64] Ramirez J. Martinez A. Lectez B. Lee S. Y. Franco M. Barrio R.. (2015). Proteomic analysis of the ubiquitin landscape in the *Drosophila* embryonic nervous system and the adult photoreceptor cells. PLoS One 10:e0139083. 10.1371/journal.pone.0139083 26460970 PMC4604154

[B65] Rieger D. Fraunholz C. Popp J. Bichler D. Dittmann R. Helfrich-Förster C. (2007). The fruit fly *Drosophila melanogaster* favors dim light and times its activity peaks to early dawn and late dusk. J. Biol. Rhythms 22, 387–399. 10.1177/0748730407306198 17876060

[B66] Rosato E. Codd V. Mazzotta G. Piccin A. Zordan M. Costa R.. (2001). Light-dependent interaction between *Drosophila* CRY and the clock protein PER mediated by the carboxy terminus of CRY. Curr. Biol. 11, 909–917. 10.1016/s0960-9822(01)00259-7 11448767

[B67] Ruby N. F. Brennan T. J. Xie X. Cao V. Franken P. Heller H. C.. (2002). Role of melanopsin in circadian responses to light. Science 298, 2211–2213. 10.1126/science.1076701 12481140

[B68] Schindelin J. Arganda-Carreras I. Frise E. Kaynig V. Longair M. Pietzsch S.. (2012). Fiji: an open-source platform for biological-image analysis. Nat. Methods 9, 676–682. 10.1038/nmeth.2019 22743772 PMC3855844

[B69] Schlichting M. Grebler R. Menegazzi P. Helfrich-Förster C. (2015). Twilight dominates over moonlight in adjusting *Drosophila’s* activity pattern. J. Biol. Rhythms 30, 117–128. 10.1177/0748730415575245 25838418

[B70] Schlichting M. Grebler R. Peschel N. Yoshii T. Helfrich-Förster C. (2014). Moonlight detection by *Drosophila’s* endogenous clock depends on multiple photopigments in the compound eyes. J. Biol. Rhythms 29, 75–86. 10.1177/0748730413520428 24682202

[B71] Schlichting M. Helfrich-Förster C. (2015). “Photic entrainment in *Drosophila* assessed by locomotor activity recordings,” in Methods in Enzymology: Circadian Rhythms and Biological Clocks, (Vol. 551) ed. Sehgal A. (Burlington, MA: Academic Press), 387–405. 10.1016/bs.mie.2014.10.017 25707274

[B72] Schmid B. Helfrich-Förster C. Yoshii T. (2011). A new ImageJ plug-in “ActogramJ” for chronobiological analyses. J. Biol. Rhythms 26, 464–467. 10.1177/0748730411414264 21921300

[B73] Selby C. P. Thompson C. Schmitz T. M. Van Gelder R. N. Sancar A. (2000). Functional redundancy of cryptochromes and classical photoreceptors for nonvisual ocular photoreception in mice. Proc. Natl. Acad. Sci. U S A 97, 14697–14702. 10.1073/pnas.260498597 11114194 PMC18981

[B74] Stanewsky R. Kaneko M. Emery P. Beretta B. Wager-Smith K. Kay S. A.. (1998). The *cry^b^* mutation identifies cryptochrome as a circadian photoreceptor in *Drosophila*. Cell 95, 681–692. 10.1016/s0092-8674(00)81638-4 9845370

[B75] Thompson C. L. Bowes Rickman C. Shaw S. J. Ebright J. N. Kelly U. Sancar A.. (2003). Expression of the blue-light receptor cryptochrome in the human retina. Invest. Ophthalmol. Vis. Sci. 44, 4515–4521. 10.1167/iovs.03-0303 14507900

[B76] Thresher R. J. Vitaterna M. H. Miyamoto Y. Kazantsev A. Hsu D. S. Petit C.. (1998). Role of mouse cryptochrome blue-light photoreceptor in circadian photoresponses. Science 282, 1490–1494. 10.1126/science.282.5393.1490 9822380

[B78] Van Gelder R. N. Wee R. Lee J. A. Tu D. C. (2003). Reduced pupillary light responses in mice lacking cryptochromes. Science 299:222. 10.1126/science.1079536 12522242

[B79] Vanin S. Bhutani S. Montelli S. Menegazzi P. Green E. W. Pegoraro M.. (2012). Unexpected features of *Drosophila* circadian behavioural rhythms under natural conditions. Nature 484, 371–375. 10.1038/nature10991 22495312

[B80] Vinayak P. Coupar J. Hughes S. E. Fozdar P. Kilby J. Garren E.. (2013). Exquisite light sensitivity of *Drosophila melanogaster* cryptochrome. PLoS Genet. 9:e1003615. 10.1371/journal.pgen.1003615 23874218 PMC3715431

[B81] Wilm M. Shevchenko A. Houthaeve T. Breit S. Schweigerer L. Fotsis T.. (1996). Femtomole sequencing of proteins from polyacrylamide gels by nano-electrospray mass spectrometry. Nature 379, 466–469. 10.1038/379466a0 8559255

[B82] Yang Z. Emerson M. Su H. S. Sehgal A. (1998). Response of the timeless protein to light correlates with behavioral entrainment and suggests a nonvisual pathway for circadian photoreception. Neuron 21, 215–223. 10.1016/s0896-6273(00)80528-0 9697865

[B83] Yoshii T. Ahmad M. Helfrich-Förster C. (2009). Cryptochrome mediates light-dependent magnetosensitivity of *Drosophila’s* circadian clock. PLoS Biol. 7:e1000086. 10.1371/journal.pbio.1000086 19355790 PMC2667543

[B84] Yoshii T. Todo T. Wülbeck C. Stanewsky R. Helfrich-Förster C. (2008). Cryptochrome is present in the compound eyes and a subset of *Drosophila’s* clock neurons. J. Comp. Neurol. 508, 952–966. 10.1002/cne.21702 18399544

[B85] Yuan Q. Metterville D. Briscoe A. D. Reppert S. M. (2007). Insect cryptochromes: gene duplication and loss define diverse ways to construct insect circadian clocks. Mol. Biol. Evol. 24, 948–955. 10.1093/molbev/msm011 17244599

[B86] Zheng X. Sehgal A. (2012). Speed control: cogs and gears that drive the circadian clock. Trends Neurosci. 35, 574–585. 10.1016/j.tins.2012.05.007 22748426 PMC3434952

[B87] Zhu H. Yuan Q. Briscoe A. D. Froy O. Casselman A. Reppert S. M. (2005). The two CRYs of the butterfly. Curr. Biol. 15, R953–R954. 10.1016/j.cub.2005.11.030 16332522

