# Supplementary material for: Cryptochrome Interacts With Actin and Enhances Eye-Mediated Light Sensitivity of the Circadian Clock in Drosophila melanogaster
Source: Front Mol Neurosci. 2018 Jul 18;11:238. doi: 10.3389/fnmol.2018.00238 (PMC6058042; doi:10.3389/fnmol.2018.00238)
Supplement: Supplementary file 1 [file Table_1.PDF]

Table S1. Nucleotide sequence of primer used.

PRIMER	SEQUENCE (5'-3')
pEG_hCRY2_F	*cggcgactggctggaattc*ATGGCGGCAACTGTGGCAACG
pEG_hCRY2_R	*ctgcaggtcgactcgag*TCAGGCATCCTTGCTCGGCAGCTC
pJG_hact_F	*gtgccagattatgcctctcccgaattc*ATGGATGATGATATCGCCGCG
pJG_hact_R	*cgaagaagtccaaagcttctcgag*CTAGAAGCATTTGCGGTGGACG
pJG_act57B_F	*tgtgccagattatgcctctcccgaattc*ATGTGTGACGATGAAGTTGCTGCTCTGGTC
pJG_act57B_R	*cgaagaagtccaaagcttctcgag*TTAGAAGCACTTGCGGTGGACGATGCCA
pJG_act5c_F	*tgtgccagattatgcctctcccgaattc*ATGTGTGACGAAGAAGTTGCTGCTCTGGTT
pJG_act5c_R	*cgaagaagtccaaagcttctcgag*TTAGAAGCACTTGCGGTGCACAATGGAG

hCRY2: NM_021117.3, hActin-Beta: NM_001101.3, dActin-5C: FBgn0000042, dActin-57B: FBgn0000044
