# Supplementary material for: Cryptochrome Interacts With Actin and Enhances Eye-Mediated Light Sensitivity of the Circadian Clock in Drosophila melanogaster
Source: Front Mol Neurosci. 2018 Jul 18;11:238. doi: 10.3389/fnmol.2018.00238 (PMC6058042; doi:10.3389/fnmol.2018.00238)
Supplement: Supplementary file 2 [file Table_2.PDF]

Table S2. Expected molecular masses of fusions used in yeast experiments.

FUSION	MM (KDa)
LexA-hCRY2	66,95
AD-hActin-Beta	53,23
AD-dActin-5C	53,23
AD-dActin-57B	53,23
