# Supplementary material for: Cryptochrome Interacts With Actin and Enhances Eye-Mediated Light Sensitivity of the Circadian Clock in Drosophila melanogaster
Source: Front Mol Neurosci. 2018 Jul 18;11:238. doi: 10.3389/fnmol.2018.00238 (PMC6058042; doi:10.3389/fnmol.2018.00238)
Supplement: Supplementary file 3 [file Table_3.PDF]

Table S3. List of the proteins identified by MALDI-TOF and LC-MS/MS analyses [a]

Spot	Protein name	Accession no. [b]	Theoretical MW (kDa)/p*I* [c]	MALDI-MS analysis			MS/MS peptides [d]
				Matched peptides	Coverage %	Score	
X1	**Actin-87E**	P10981	41.6/5.30	13	50	96	3[e]
	**Actin-5C**	P10987	41.6/5.30	11	47	75	
X2	**Actin-57B**	P53501	41.6/5.23	11	46	84	2

[a] For the spots X1 and X2, the identified proteins with their accession numbers and theoretical molecular weights/p*I* are listed. Protein identification was performed with the MASCOT software searching MS data against the sequences of *Drosophila* of the Swiss-Prot database. [b] Accession no. from the UniProtKB database. [c] Theoretical MW and p*I* were calculated using the Compute pI/MW tool available on the ExPASy website (www.expasy.org). [d] Peptides sequenced by LC-MS/MS that matched to the protein with a significant score ($p<0.05$). [e] The peptides sequenced by LC-MS/MS are common to both Actin-87E and Actin-5C.
