# Supplementary material for: Cryptochrome Interacts With Actin and Enhances Eye-Mediated Light Sensitivity of the Circadian Clock in Drosophila melanogaster
Source: Front Mol Neurosci. 2018 Jul 18;11:238. doi: 10.3389/fnmol.2018.00238 (PMC6058042; doi:10.3389/fnmol.2018.00238)
Supplement: Supplementary file 4 [file Data_Sheet_1.PDF]

Cryptochrome interacts with actin and enhances eye-mediated light sensitivity of the circadian clock in *Drosophila melanogaster*

Matthias Schlichting[a,b], Dirk Rieger[a], Paola Cusumano[c], Rudi Grebler[a], Rodolfo Costa[c], Gabriella M. Mazzotta[c#], Charlotte Helfrich-Förster[a#]

SUPPLEMENTARY INFORMATION

Methods

Yeast-Two Hybrid

Bait construction._The full length coding sequence of dCRY was cloned in the bait vector pDBLeu (derivative of pPC97_ Chevray and Nathans (1992)), in frame with the DNA binding domain of Gal4 (aa1-147; GAL4-DB), using standard molecular biology techniques. The pDB-CRY construct was transformed in Mav203 and checked for correct expression with a specific rabbit anti-dCRY antibody (1:1000_Neosystem Laboratoire, Strasburg, France, aa 76-90_ DGRGRLLVFEGEPAY).

Library screening. The expression library, kindly provided by Dr. S. Goodwin, University of Oxford, UK, was prepared from cDNA of wild-type *Drosophila* heads (Canton S strain) cloned in pPC86 vector (Life Technologies®). The Mav203 strain containing the bait construct (Mav-dCRY1) was transformed with the cDNA library according to the modified procedure of the lithium acetate method (Gietz et al., 1992). The transformants were then tested for *LacZ* reporter gene expression the filter assay according to Breeden and Nasmyth (1985). Positive clones were isolated from the yeast strain and re-transformed in the Mav-dCRY for further test. Clones that resulted positive at this further screening were sequenced with primers pPC86_F: 5'-TATAACGCGTTTGGAATCACT-3' and pPC86_R: 5'-GTAAATTTCTGACGAGGTAGAC-3', designed on the plasmid sequence. Quantitative CPRG assay was performed according to ProQUESTTM Two-Hybrid System manual (LIFE TECHNOLOGIES®). Statistic analysis was performed with Graphpad Prism v4 using one-way ANOVA followed by Tukey's multiple comparisons test.

Co Immunoprecipitation and 2D SDS PAGE

Three to five days old flies overexpressing HAdCRY (Dissel et al., (2004); yw; tim-GAL4/+; UAS-Hacry/+) were collected at ZT24 (ZT 0 lights-on and ZT 12 lights-off in a 12:12 light–dark cycle). Heads were homogenized in extraction buffer [20 mM Hepes, pH 7.5, 100 mM KCl, 2.5 mM EDTA, pH 8, 5% glycerol, 0.5% Triton X-100, 1 mM DTT, complete protease inhibitors (Roche)], centrifuged at maximum speed for 10 min and the supernatant pre-cleared with protein-G agarose beads (Sigma) for 20 min. The extract was then incubated with anti-HA (1:1000, Sigma) for 2 h at 4°C before the addition of 30 µl of protein G agarose beads (1:1 slurry) for 1 h. The beads were precipitated by centrifugation at 2000g and then washed three times with 1 ml of extraction buffer and once with 1 ml of 20 mM Hepes, pH 7.5.

The 2D electrophoresis has been performed according to Khoudoli et al., (2004), with some modifications. Protein complexes were solubilized by heat treatment (5 min at 95°C) in presence of 100mM DTT and 0.2% SDS, precipitated in 80% acetone at -20°C and solubilized for 6 hours in resuspension buffer (30 mM Tris Base, 7 M Urea, 2 M Thiourea, 1.2% CHAPS, 0.14% ASB14, 0.25% Ampholytes, 43 mM DTT), with the addition of 60 mM Acrylamide after 3 hours, in order to alkylate the proteins (Mineki et al., 2002). Isoelectric focusing (IEF) was performed in 7 cm IPG strips of pH range 4–7 (ReadyStrip™_Bio-rad); strips have been passively rehydrated for 16 hours and then iso-electro focused by a two-phase protocol: 30 min at 250 V, 3 h and 30 min at 5500 V and 500 V until the complete

focusing. After IEF, strips were equilibrated in Equilibration buffer (50 mM Bis-Tris pH 6.4, 6 M Urea, 30% (w/v) glycerol, 2% SDS) containing 50 mM DTT for 20 min and 360 mM Acrylamide for further 20 min. Strips were then placed on a 4-12 % pre-cast "ZOOM NuPAGE gel" (Invitrogen®) with the help of a 0.5% agarose matrix and and run at room temperature at 50 V.

Protein identification by mass spectrometry. After the separation of proteins on the gel, Coomassie-stained protein spots were excised and in-gel digested, as previously described (Wilm et al., 1996). Briefly, gel pieces were destained and the proteins digested with porcine trypsin (modified sequencing grade; Promega, Madison, WI, USA) overnight at 37 °C. The supernatants were then transferred to other tubes and residual tryptic peptides were extracted upon incubation of gel spots with 25 mm NH_4HCO_3 at 37 °C for 15 min followed by shrinking of gel pieces with acetonitrile, and then upon incubation with 5% (v/v) formic acid at 37 °C for 15 min followed by shrinking with acetonitrile. The extracts were combined with the primary supernatant and dried in a SpeedVac centrifuge (Savant Instrument Inc., NY, USA). LC-MS/MS analyses were performed on protein digests dissolved in 0.1% trifluoroacetic acid, 5% acetonitrile and using a Micromass CapLC unit (Waters) interfaced to a Micromass Q-Tof Micro mass spectrometer (Waters) equipped with a nanospray source. Tryptic digests were loaded at a flow rate of 15 µl/min onto an Atlantis dC18 Trap Column. After valve switching, the sample was separated on a Symmetry C18 column (150 x 0.075 mm, 3.5 µm particle size) (Waters) at a flow rate of 3.5 µl/min using a gradient from 1% B to 40% B in 43 min and from 40% to 70% B% in 7 min (solvent A: 95% H2O, 5% acetonitrile, 0.1% formic acid; solvent B: 5% H2O, 95% acetonitrile, 0.1% formic acid). Instrument control, data acquisition and processing were achieved with MassLynx V4.1 software (Waters). MALDI mass spectrometry measurements were performed on a MALDI-TOF Ultraflex II (Bruker Daltonics, Bremen, Germany) operating in the positive-ion reflectron mode. A saturated solution of a-cyano-4hydroxycinnamic acid in water, 0.1% TFA/acetonitrile (1/1 v/v ratio) was used as matrix and mixed at a v/v ratio of 1:1 with the digests dissolved in 0.5% TFA aqueous solution. MALDI-TOF and LC-MS/MS data were analyzed by the online MASCOT software (Matrix Science, http://www.matrixscience.com) against the *Drosophila* (fruit flies) sequences of the Swiss-Prot database (release 2012_04). The following parameters were used in the MASCOT search: trypsin specificity; maximum number of missed cleavages: 1; fixed modification: propionamide (Cys); variable modifications: oxidation (Met); peptide mass tolerance: ± 0.2 Da; (fragment mass tolerance: ± 0.5 Da for the MS/MS data); protein mass: unrestricted; mass values: monoisotopic.

References

Breeden L, Nasmyth K. 1985. Regulation of the yeast HO gene. Cold Spring Harbor Symp. Quant. Biol. 50, 643-650.
Chevray PM, Nathans D. 1992. Protein interaction cloning in yeast: identification of mammalian proteins that react with the leucine zipper of Jun. Proc. Nat. Acad. Sci. U. S. A. 89, 5789-5793.
Dissel S, Codd V, Fedic R, Garner KJ, Costa R, Kyriacou CP, Rosato E. 2004. A constitutively active cryptochrome in *Drosophila melanogaster*. Nature Neurosci. 7, 834-840. 10.1038/nn1285.
Gietz D, Jean AS, Woods RA, Schiestl RH. 1992. Improved method for high efficiency transformation of intact yeast cells. Nucleic Acids Research 20, 1425.
Khoudoli GA, Porter IM, Blow JJ, Swedlow JR. 2004. Optimisation of the two-dimensional gel electrophoresis protocol using the taguchi approach. Proteome Science 2, 6.

Mineki R, Taka H, Fujimura T, Kikkawa M, Shindo N, Murayama K. 2002. In situ alkylation with acrylamide for identification of cysteinyl residues in proteins during one- and two-dimensional sodium dodecyl sulphate-polyacrylamide gel electrophoresis. Proteomics 2, 1672-1681.
Wilm M, Shevchenko A, Houthaeve T, Breit S, Schweigerer L, Fotsis T, Mann M. 1996. Femtomole sequencing of proteins from polyacrylamide gels by nano-electrospray mass spectrometry. Nature 379, 466-469. 10.1038/379466a0.
