# Supplementary material for: Cryptochrome Interacts With Actin and Enhances Eye-Mediated Light Sensitivity of the Circadian Clock in Drosophila melanogaster
Source: Front Mol Neurosci. 2018 Jul 18;11:238. doi: 10.3389/fnmol.2018.00238 (PMC6058042; doi:10.3389/fnmol.2018.00238)
Supplement: Supplementary file 5 [file Image_1.pdf]

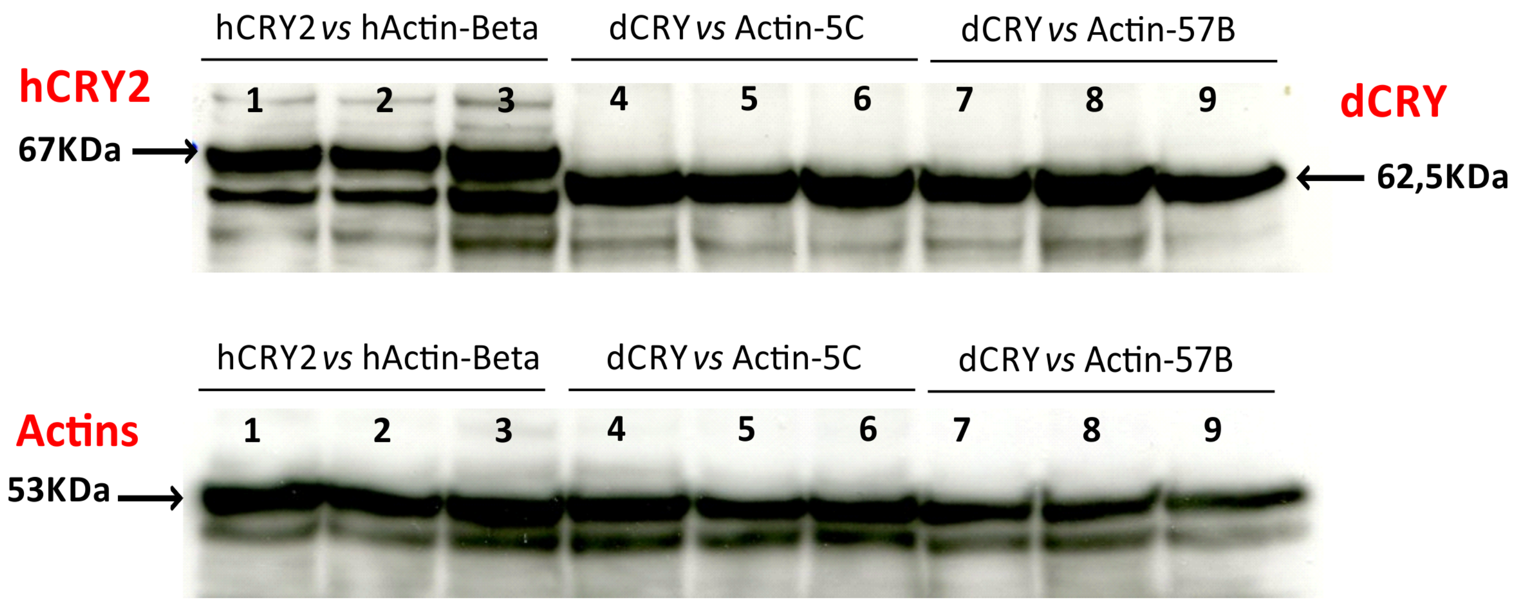

Figure S1. Western blot analysis of independent yeast clones tested for bait and prey fusions expression.
Three independent clones, randomly chosen among the 7 tested, have been analyzed for the expression of bait (CRYs) and prey (Actins) fusions. After SDS-PAGE, the same membrane has been probed with anti-LexA and anti-HA antibodies, to reveal bait and prey fusions, respectively. The signals corresponding to the fusions are shown.
