# Supplementary material for: Cryptochrome Interacts With Actin and Enhances Eye-Mediated Light Sensitivity of the Circadian Clock in Drosophila melanogaster
Source: Front Mol Neurosci. 2018 Jul 18;11:238. doi: 10.3389/fnmol.2018.00238 (PMC6058042; doi:10.3389/fnmol.2018.00238)
Supplement: Supplementary file 6 [file Image_2.pdf]

```
Actin-79B         MCDEEASALVVDNGSGMCKAGFAGDDAPRAVFPSIVGRPRHQGVMVGMGQKDCYVGDEAQ 60
Actin-88F         MCDDDAGALVIDNGSGMCKAGFAGDDAPRAVFPSIVGRPRHQGVMVGMGQKDSYVGDEAQ 60
Actin-57B         MCDDEVAALVVDNGSGMCKAGFAGDDAPRAVFPSIVGRPRHQGVMVGMGQKDSYVGDEAQ 60
Actin-87E         MCDDEVAALVVDNGSGMCKAGFAGDDAPRAVFPSIVGRPRHQGVMVGMGQKDSYVGDEAQ 60
Actin-5C          MCDEEVAALVVDNGSGMCKAGFAGDDAPRAVFPSIVGRPRHQGVMVGMGQKDSYVGDEAQ 60
Actin-42A         MCDEEVAALVVDNGSGMCKAGFAGDDAPRAVFPSIVGRPRHQGVMVGMGQKDSYVGDEAQ 60
                  ***::..***:*****************************************.*******

Actin-79B         SKRGILSLKYPIEHGIITNWDDMEKVWHHTFYNELRVAPEEHPVLLTEAPLNPKANREKM 120
Actin-88F         SKRGILTLKYPIEHGIITNWDDMEKIWHHTFYNELRVAPEEHPVLLTEAPLNPKANREKM 120
Actin-57B         SKRGILTLKYPIEHGIITNWDDMEKIWHHTFYNELRVAPEEHPVLLTEAPLNPKANREKM 120
Actin-87E         SKRGILTLKYPIEHGIITNWDDMEKIWHHTFYNELRVAPEEHPVLLTEAPLNPKANREKM 120
Actin-5C          SKRGILTLKYPIEHGIVTNWDDMEKIWHHTFYNELRVAPEEHPVLLTEAPLNPKANREKM 120
Actin-42A         SKRGILTLKYPIEHGIVTNWDDMEKIWHHTFYNELRVAPEEHPVLLTEAPLNPKANREKM 120
                  ******:*********:********:**********************************

Actin-79B         TQIMFETFNSPAMYVAIQAVLSLYASGRTTGIVLDSGDGVSHTVPIYEGYALPHAILRLD 180
Actin-88F         TQIMFETFNSPAMYVAIQAVLSLYASGRTTGIVLDSGDGVSHTVPIYEGFALPHAILRLD 180
Actin-57B         TQIMFETFNSPAMYVAIQAVLSLYASGRTTGIVLDSGDGVSHTVPIYEGYALPHAILRLD 180
Actin-87E         TQIMFETFNAPAMYVAIQAVLSLYASGRTTGIVLDSGDGVSHTVPIYEGYALPHAILRLD 180
Actin-5C          TQIMFETFNTPAMYVAIQAVLSLYASGRTTGIVLDSGDGVSHTVPIYEGYALPHAILRLD 180
Actin-42A         TQIMFETFNTPAMYVAIQAVLSLYASGRTTGIVLDSGDGVSHTVPIYEGYALPHAILRLD 180
                  *********:***************************************:**********

Actin-79B         LAGRDLTDYLMKILTERGYSFTTTAEREIVRDIKEKLCYVALDFEQEMATAAASTSLEKS 240
Actin-88F         LAGRDLTDYLMKILTERGYSFTTTAEREIVRDIKEKLCYVALDFEQEMATAAASTSLEKS 240
Actin-57B         LAGRDLTDYLMKILTERGYSFTTTAEREIVRDIKEKLCYVALDFEQEMATAAASTSLEKS 240
Actin-87E         LAGRDLTDYLMKILTERGYSFTTTAEREIVRDIKEKLCYVALDFEQEMATAAASTSLEKS 240
Actin-5C          LAGRDLTDYLMKILTERGYSFTTTAEREIVRDIKEKLCYVALDFEQEMATAASSSSLEKS 240
Actin-42A         LAGRDLTDYLMKILTERGYSFTTTAEREIVRDIKEKLCYVALDFEQEMATAASSSSLEKS 240
                  ****************************************************:*:*****

Actin-79B         YELPDGQVITIGNERFRTPEALFQPSFLGMESCGIHETVYQSIMKCDVDIRKDLYANNVL 300
Actin-88F         YELPDGQVITIGNERFRCPEALFQPSFLGMESCGIHETVYNSIMKCDVDIRKDLYANSVL 300
Actin-57B         YELPDGQVITIGNERFRCPESLFQPSFLGMESCGIHETVYNSIMKCDVDIRKDLYANIVM 300
Actin-87E         YELPDGQVITIGNERFRCPESLFQPSFLGMESCGIHETVYNSIMKCDVDIRKDLYANIVM 300
Actin-5C          YELPDGQVITIGNERFRCPEALFQPSFLGMEACGIHETTYNSIMKCDVDIRKDLYANTVL 300
Actin-42A         YELPDGQVITIGNERFRCPESLFQPSFLGMEACGIHETTYNSIMKCDVDIRKDLYANTVL 300
                  ***************** **:**********:******.*:**************** *:

Actin-79B         SGGTTMYPGIADRMQKEITALAPSTIKIKIIAPPERKYSVWIGGSILASLSTFQQMWISK 360
Actin-88F         SGGTTMYPGIADRMQKEITALAPSTIKIKIIAPPERKYSVWIGGSILASLSTFQQMWISK 360
Actin-57B         SGGTTMYPGIADRMQKEITSLAPSTIKIKIIAPPERKYSVWIGGSILASLSTFQQMWISK 360
Actin-87E         SGGTTMYPGIADRMQKEITALAPSTIKIKIIAPPERKYSVWIGGSILASLSTFQQMWISK 360
Actin-5C          SGGTTMYPGIADRMQKEITALAPSTMKIKIIAPPERKYSVWIGGSILASLSTFQQMWISK 360
Actin-42A         SGGTTMYPGIADRMQKEITALAPSTMKIKIVAPPERKYSVWIGGSILASLSTFQQMWISK 360
                  ******************:*****:****:******************************

Actin-79B         QEYDESGPGIVHRKCF 376
Actin-88F         QEYDESGPGIVHRKCF 376
Actin-57B         EEYDESGPGIVHRKCF 376
Actin-87E         QEYDESGPGIVHRKCF 376
Actin-5C          QEYDESGPSIVHRKCF 376
Actin-42A         QEYDESGPSIVHRKCF 376
                  :*******.*******
```

Figure S2. Clustal W alignment of proteins encoded by six actin genes in *Drosophila*.

***** identical residues in all sequences, **:** highly conserved residues, **.** weakly conserved residues. Actin-79B is encoded by gene CG7478 on the left arm of chromosome 3, Actin-88F by gene CG5178 on the right arm of chromosome 3, Actin-57B by gene CG10067 on the right arm of chromosome 2, Actin-87E by gene CG18290 on the right arm of chromosome 3, Actin-5C by gene CG4027 on the X-chromosome and Actin-42A by gene CG12051 on the right arm of chromosome 2.
