# Supplementary material for: Cryptochrome Interacts With Actin and Enhances Eye-Mediated Light Sensitivity of the Circadian Clock in Drosophila melanogaster
Source: Front Mol Neurosci. 2018 Jul 18;11:238. doi: 10.3389/fnmol.2018.00238 (PMC6058042; doi:10.3389/fnmol.2018.00238)
Supplement: Supplementary file 7 [file Image_3.PDF]

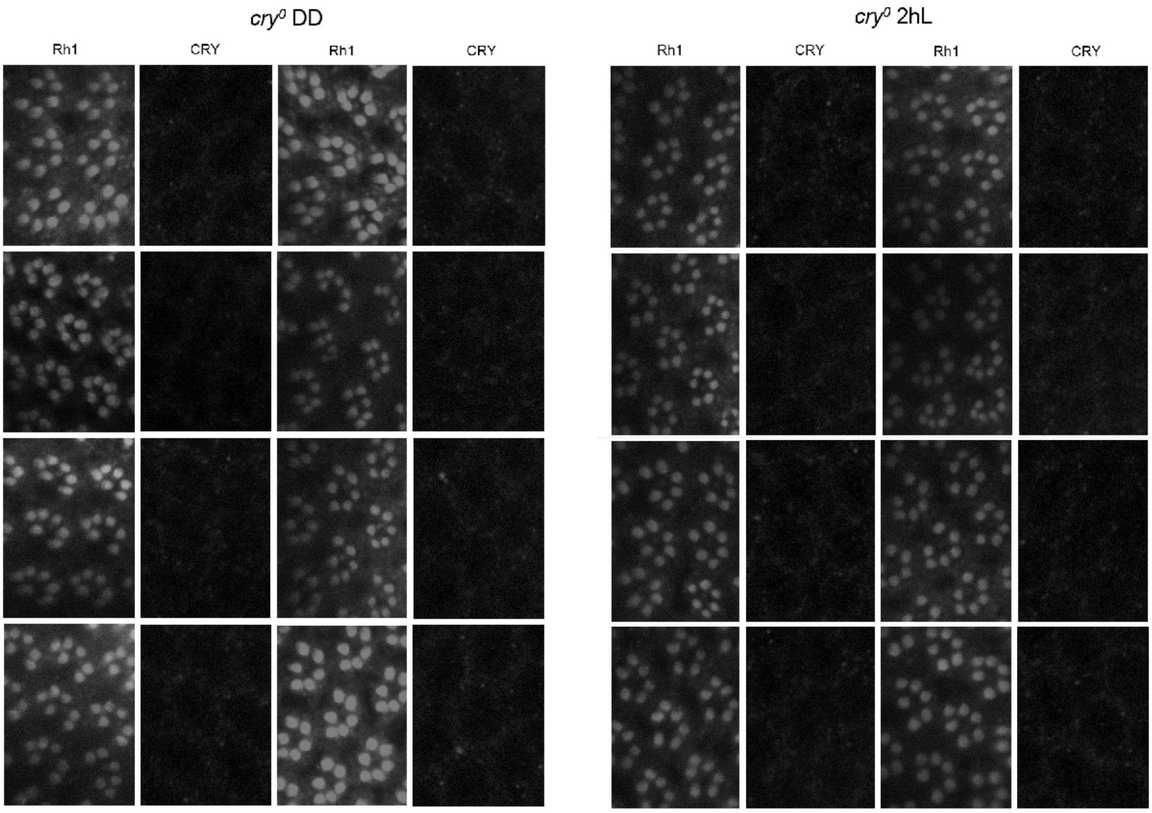

Figure S3. CRY and Rhodopsin 1 (Rh1) staining in the retina of *cry^{01}* mutants.

CRY is neither visible under constant darkness (DD) nor after a 2 hour light-exposure (2h L).
