# Supplementary material for: Cryptochrome Interacts With Actin and Enhances Eye-Mediated Light Sensitivity of the Circadian Clock in Drosophila melanogaster
Source: Front Mol Neurosci. 2018 Jul 18;11:238. doi: 10.3389/fnmol.2018.00238 (PMC6058042; doi:10.3389/fnmol.2018.00238)
Supplement: Supplementary file 8 [file Image_4.PDF]

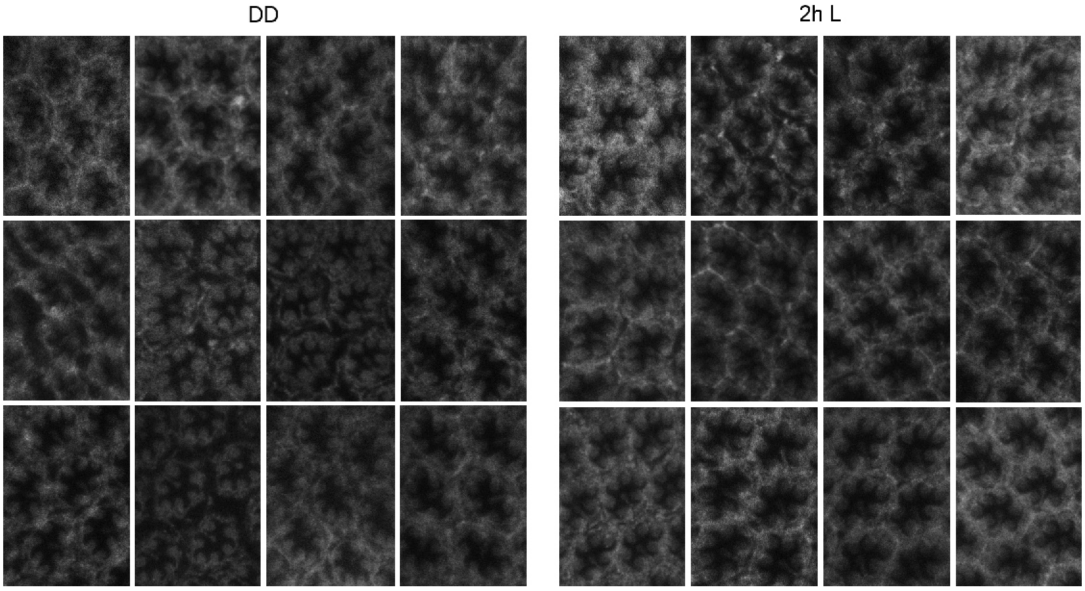

Figure S4. CRY staining in the retina of wildtype flies.

12 retinas of flies kept under constant darkness (DD) and after a 2 hour light-exposure (2h L) are shown, respectively. CRY staining intensity remains the same after the 2 hour light-exposure.
