# Supplementary material for: Cryptochrome Interacts With Actin and Enhances Eye-Mediated Light Sensitivity of the Circadian Clock in Drosophila melanogaster
Source: Front Mol Neurosci. 2018 Jul 18;11:238. doi: 10.3389/fnmol.2018.00238 (PMC6058042; doi:10.3389/fnmol.2018.00238)
Supplement: Supplementary file 9 [file Image_5.PDF]

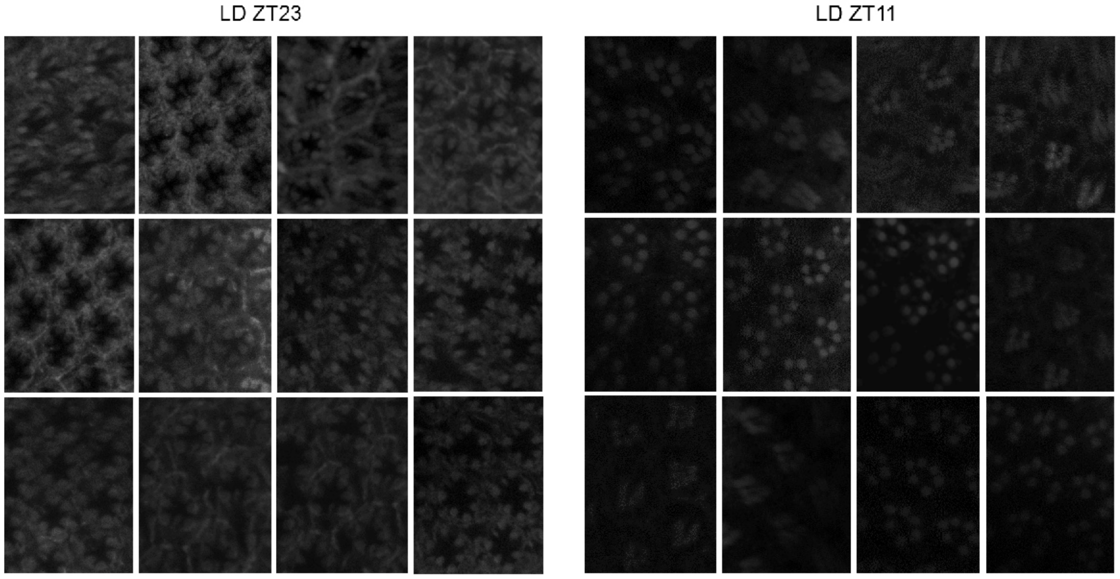

Figure S5. CRY staining in the retina of wildtype flies kept under regular 12:12 h light-dark cycles.

12 retinas of flies killed one hour before lights-on (ZT23) and one hour before lights-off (ZT11) are shown, respectively. Note that cytoplasmic CRY staining is higher in the flies that were stained at ZT23, whereas CRY in the rhabdomeres stays about the same (see Fig. 2 in the main manuscript).
