# Supplementary material for: Cryptochrome Interacts With Actin and Enhances Eye-Mediated Light Sensitivity of the Circadian Clock in Drosophila melanogaster
Source: Front Mol Neurosci. 2018 Jul 18;11:238. doi: 10.3389/fnmol.2018.00238 (PMC6058042; doi:10.3389/fnmol.2018.00238)
Supplement: Supplementary file 10 [file Image_6.PDF]

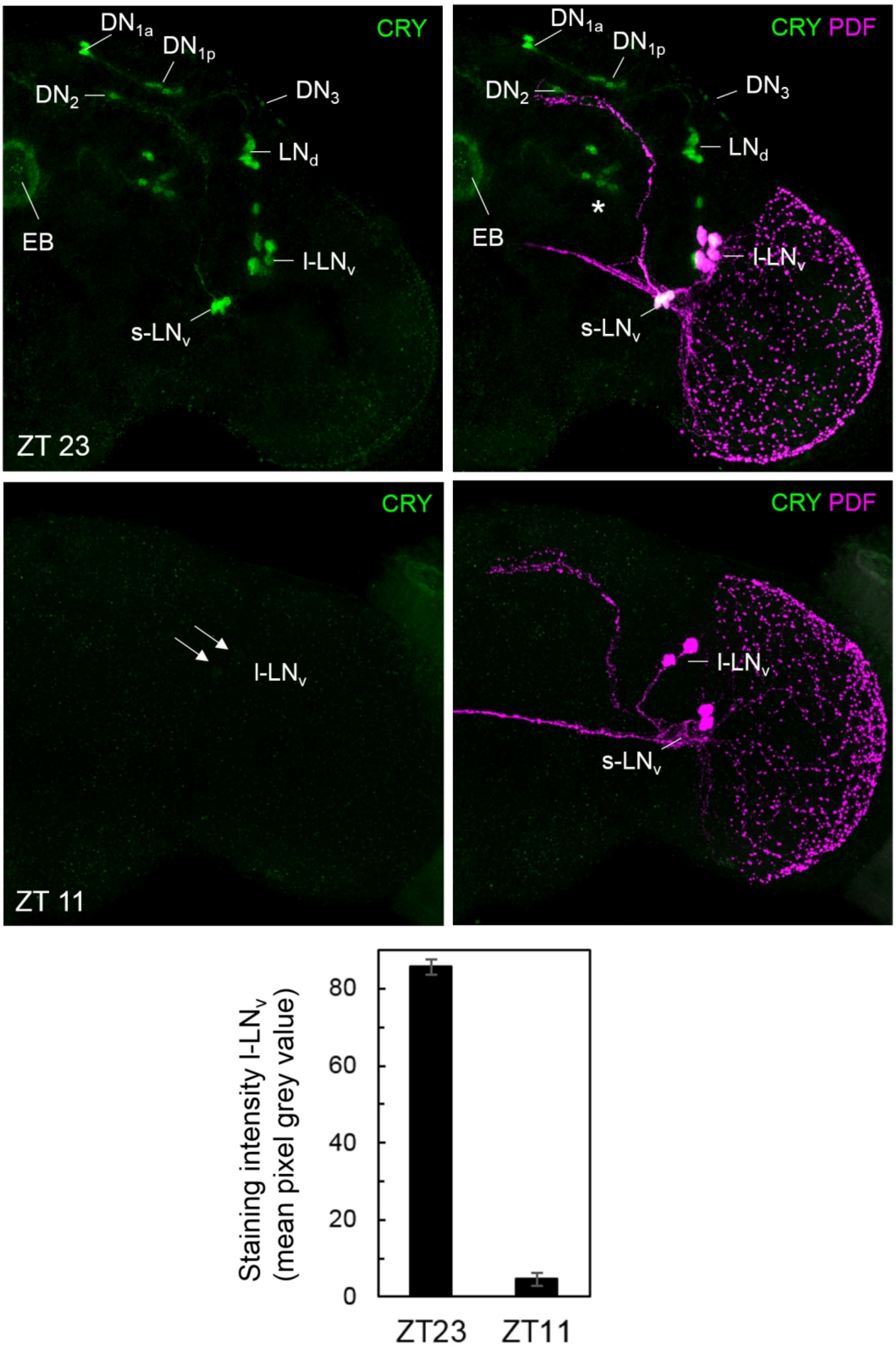

Figure S6. CRY staining in the brain of wildtype flies kept under regular 12:12 h light-dark cycles.

Representative right brain hemispheres are shown at ZT23 and ZT11. An antibody against Pigment-dispersing Factor (PDF) was used to localize the small and large ventrolateral neurons (s-LN_v and l-LN_v) and to determine CRY staining intensity in the l-LN_v (see diagram below the images). At ZT23, CRY is present in all clock neurons (LN_d = dorsolateral neurons, DN_{1a} = anterior dorsal neurons 1, DN_{1p} = posterior dorsal neurons 1, DN_2 = dorsal neurons 2) and in ellipsoid body (EB) neurons (asterisks). At ZT11 virtually no CRY staining is observed. The arrows point to weak staining in the l-LN_v.
