# Supplementary material for: Cryptochrome Interacts With Actin and Enhances Eye-Mediated Light Sensitivity of the Circadian Clock in Drosophila melanogaster
Source: Front Mol Neurosci. 2018 Jul 18;11:238. doi: 10.3389/fnmol.2018.00238 (PMC6058042; doi:10.3389/fnmol.2018.00238)
Supplement: Supplementary file 11 [file Image_7.PDF]

ZT23

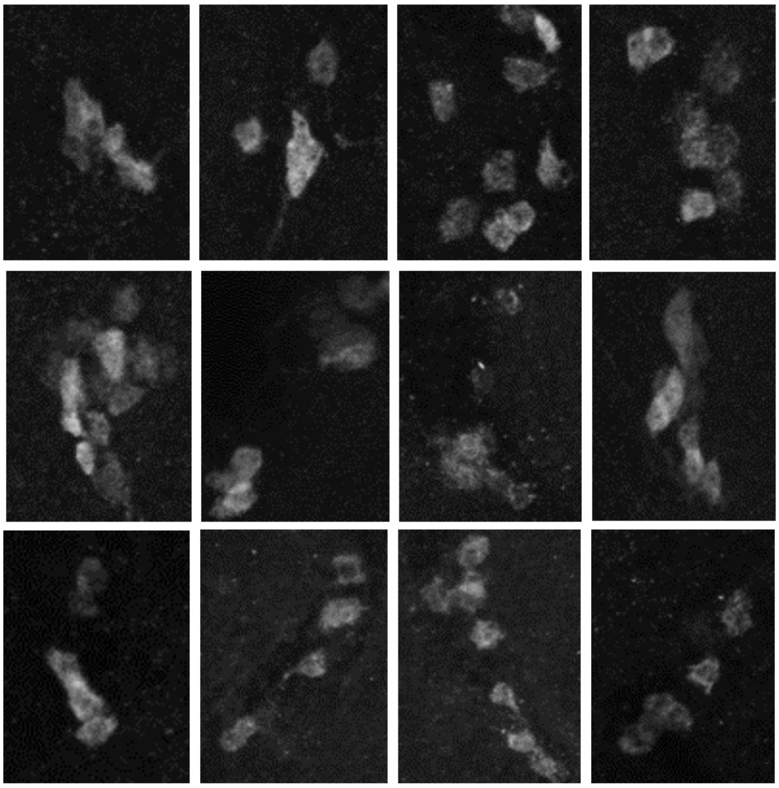

ZT11

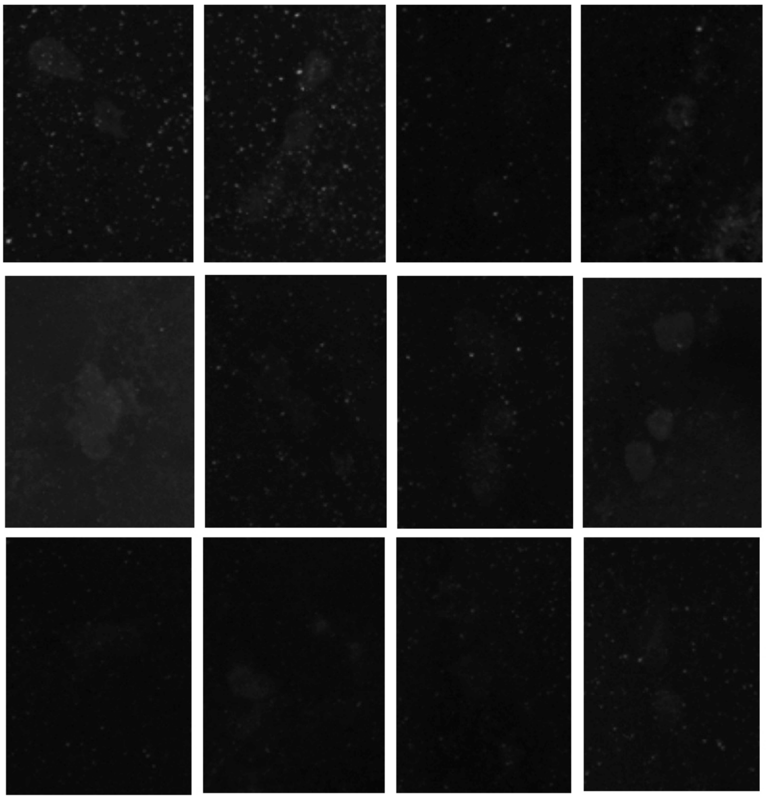

Figure S7. CRY staining in the ventrolateral neurons (LN_vs) at ZT23 and ZT11.

The staining is shown for 12 brain hemispheres, respectively.
