# Supplementary material for: Cryptochrome Interacts With Actin and Enhances Eye-Mediated Light Sensitivity of the Circadian Clock in Drosophila melanogaster
Source: Front Mol Neurosci. 2018 Jul 18;11:238. doi: 10.3389/fnmol.2018.00238 (PMC6058042; doi:10.3389/fnmol.2018.00238)
Supplement: Supplementary file 12 [file Image_8.pdf]

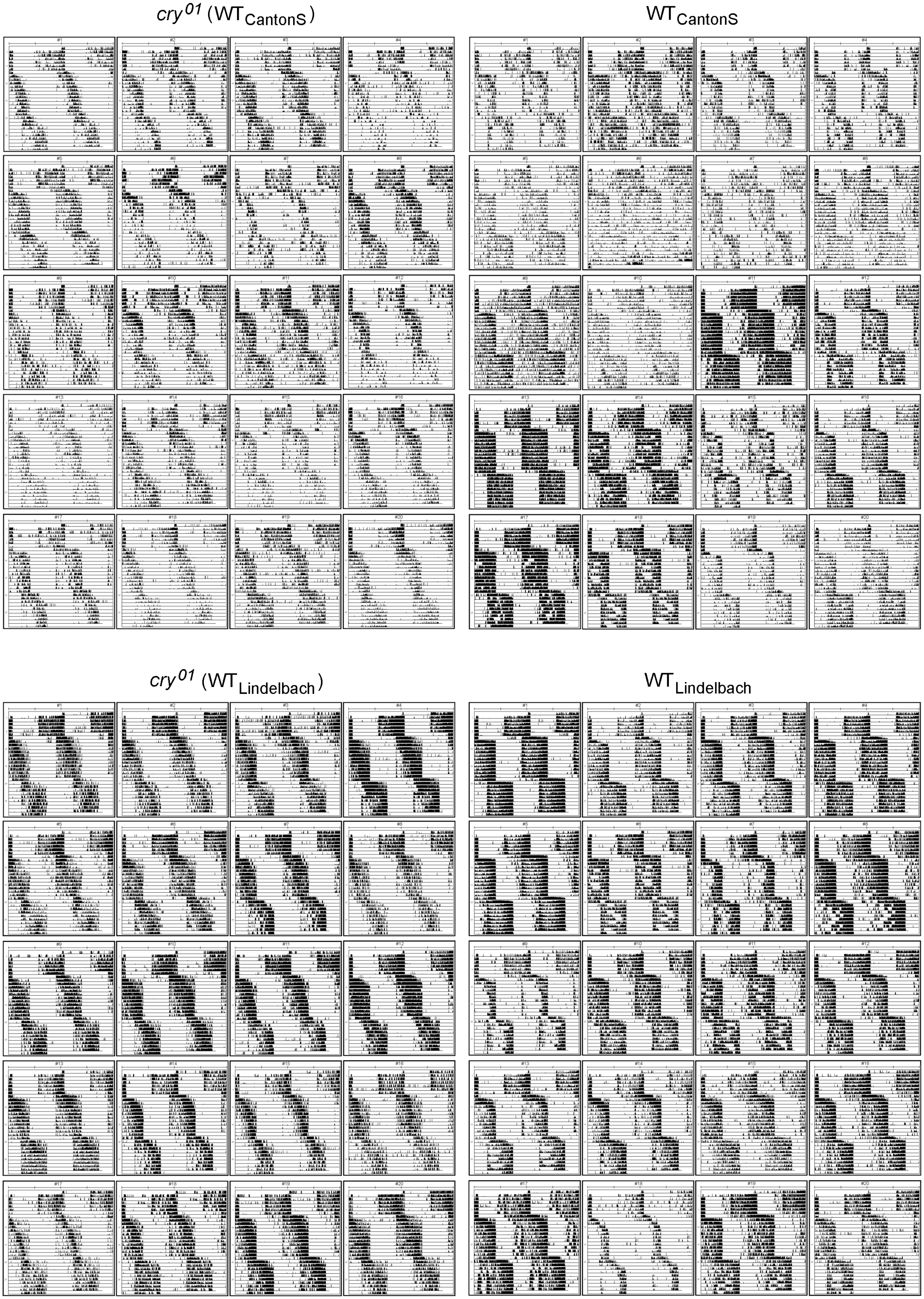

Figure S8. Individual actograms of wildtype flies and *cry*^{01} mutants in two different backgrounds (CantonS and Lindelbach) that were subjected to a 8h shift of a 12:12h red light dark cycle.
