# Supplementary material for: Cryptochrome Interacts With Actin and Enhances Eye-Mediated Light Sensitivity of the Circadian Clock in Drosophila melanogaster
Source: Front Mol Neurosci. 2018 Jul 18;11:238. doi: 10.3389/fnmol.2018.00238 (PMC6058042; doi:10.3389/fnmol.2018.00238)
Supplement: Supplementary file 13 [file Image_9.pdf]

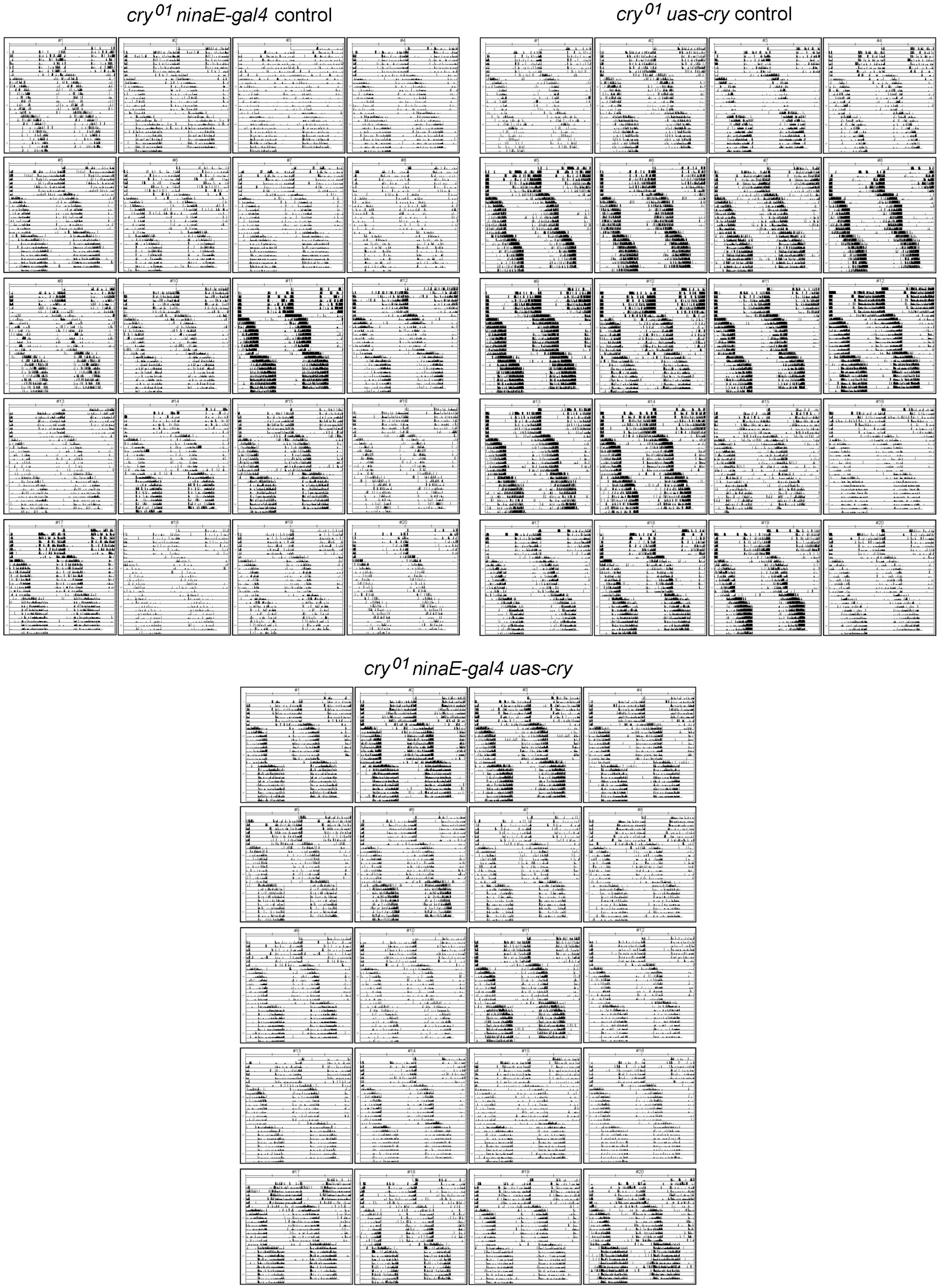

Figure S9. Individual actograms of *cry*^{01} mutant controls (upper two panels) and *cry*^{01} mutants, in which CRY was rescued in photoreceptors 1 to 6 (lower panel).
